# Reduced Thermal Conductivity and Improved Stability by B-Site Doping in Tin Halide Perovskites

**DOI:** 10.1021/acs.jpclett.4c02618

**Published:** 2025-01-06

**Authors:** Weidong Tang, Siyuan Zhang, Tianjun Liu, Chanwon Jung, Se-Ho Kim, Christina Scheu, Shengying Yue, Oliver Fenwick

**Affiliations:** † School of Engineering and Materials Science, Queen Mary University of London, Mile End Road, London E1 4NS, U.K.; ‡ Max-Planck-Institut für Eisenforschung, Max-Planck-Str. 1, 40237 Düsseldorf, Germany; § Department of Materials Science and Engineering, Korea University, Seoul 02841, Republic of Korea; ∥ State Key Laboratory for Strength and Vibration of Mechanical Structures, School of Aerospace, Xi’an Jiaotong University, Xi’an 710049, China

## Abstract

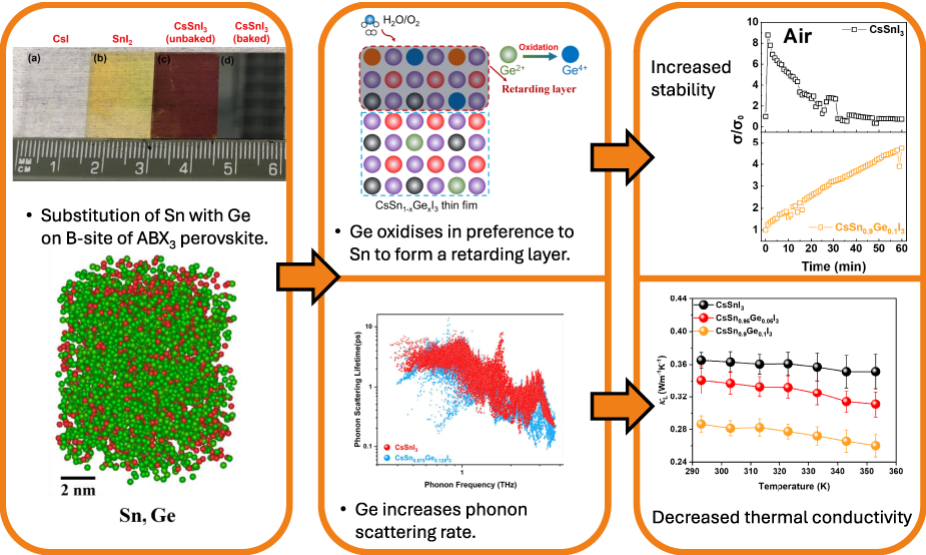

Halide perovskites have attracted recent attention as thermoelectric materials due to their low thermal conductivity combined with good charge transport characteristics. The tin halide perovskites hold the highest *zT* within metal halide perovskites and offer lower toxicity than lead-containing perovskites that are well-known for photovoltaics. In this study, we partially substitute Sn (II) with Ge (II) to form mixed metal CsSn_1–*x*_Ge_*x*_I_3_ perovskite thin films that have substantially improved stability, remaining in the black orthorhombic phase after hours of ambient air exposure. We find Ge (II) at the surface seems to be oxidized in preference to Sn (II), and this retards oxidation of the bulk of the film. Moreover, Ge substitutions dramatically reduce the lattice thermal conductivity to 0.26 ± 0.01 Wm^–1^K^–1^ for CsSn_0.9_Ge_0.1_I_3_ at 353 K. Density functional theory simulations show that Ge-doped Sn perovskites possess more low-frequency phonon modes than pristine CsSnI_3_, which leads to stronger scattering among the acoustic phonons, resulting in lower phonon group velocity and reduced phonon lifetime. These findings make an important contribution to our understanding of the origin of the reduced lattice thermal conductivity and improved electrical stability of B-site doped perovskite materials.

Due to the massive use of nonrenewable energy sources to meet economic growth, global temperatures have already rapidly risen, causing many environmental problems such as rising sea levels, and even more extreme weather. To address these environmental issues, various countries have set net zero carbon emission targets of bringing all carbon dioxide emissions to net zero by the middle of the 21st century.^1^ Currently, the main energy supply comes from burning fossil fuels where only about 30% of this energy is used and about 70% is lost in the form of waste heat.^2^ If this waste heat is utilized, emissions could be significantly reduced. Thermoelectric technology can directly convert waste heat into electricity through the Seebeck effect, which is the ability of a metal or semiconductor to develop a potential difference across its ends when placed in a temperature gradient. The thermoelectric conversion efficiency is described by the thermoelectric figure of merit, *zT* = *S*^2^*σT*/κ, where *S* is Seebeck coefficient, σ is electrical conductivity, *T* is temperature, and κ is thermal conductivity.

Metal halide perovskites (MHPs) have generated considerable recent research interest in the fields of solar cells,^3−5^ photodetectors,^6,7^ and light emitting diodes^8−10^ due to their tunable bandgap, high absorption coefficient, and long charge-carrier lifetimes.^11,12^ Recently, MHPs have also attracted widespread interest in the field of thermoelectrics because of their considerable Seebeck coefficients,^13−15^ low lattice thermal conductivity^16^ and reasonable charge mobility.^17,18^ Compared to state-of-the-art thermoelectric materials, MHPs can be fabricated as flexible thin films, and their preparation is low-cost, which makes them good candidates for thermoelectric applications at low temperatures.^19^ Furthermore, MAPbI_3_ and MASnI_3_ perovskites (MA = methylammonium CH_3_NH_3_) are predicted to have *zT* between 1 and 2,^20^ while the inorganic MHP CsSnBr_3_ is predicted to have a *zT* of 1.0.^21^ Similar values are predicted for mixed halide perovskites, such as CsPb(I_1–x_Br_*x*_)_3_ with predicted *zT* = 1.7.^22^ Meanwhile, the low-dimensional perovskite analogue, Cs_3_Cu_2_I_5_, is predicted to have *zT* = 2.6 at 600 K,^23^ a value that rivals state of the art thermoelectric materials such as SnSe and PbTe on figure of merit.

For Sn-based halide perovskites, their high conductivity leads to a significantly higher power factor (PF) and *zT* compared to Pb-based perovskites.^24−26^ In 2015, Mettan et al. estimated, from combining experimental data and different literature sources, that the thermoelectric performance of MASnI_3_ (MA = methylammonium) should be very promising.^27^ Tin-based perovskites are highly air-sensitive due to the oxidation of Sn^2+^ to Sn^4+^, which occurs rapidly because of the low redox potential of Sn^2+^.^28^ However, this oxidation process releases free charge carriers (holes) which can effectively p-dope the material to charge carrier concentrations required for thermoelectric applications. By controlling the oxidation of Sn^2+^ to Sn^4+^ in a thin surface-layer, our previous work achieved a *zT* of 0.14 (at 343 K) for mixed halide CsSnI_3–x_Cl_*x*_ perovskite thin films (x = 0.01).^29^ Yang et al. developed quasi-2D crystal structures in Sn-based halide perovskites, which enhanced both the Seebeck coefficient and the electrical conductivity, resulting in a PF of 111 μW m^–1^ K^–2^.^30^ Zheng et al. investigated the thermoelectric performance of FASnI_3_ thin films (FA = formamidinium) by introducing 2,3,5,6-tetrafluoro-7,7,8,8-tetracyanoquinodimethane (F4-TCNQ) as a dopant and obtained *zT* = 0.19.^31^ However, the low standard redox potential of Sn (II) leads to easy oxidation of Sn^2+^, resulting in the instability of Sn-based MHPs.^32^ Along with the mismatch between theoretically predicted and experimentally achieved performance, and a lack of studies on MHP thermoelectric devices, the main issue facing MHPs for thermoelectrics is their stability. Only a small number of studies have so far explored mechanisms to stabilize thermoelectric performance in Sn-MHPs, focusing on graded composition and alloying on the B- or X-site,^29,33^ including with Ge. The small number of studies in this area indicate a strong interplay between stabilization strategy and thermoelectric performance that needs detailed characterization, while there is also a lack of understanding of the role of alloying of MHPs on their thermal conductivity. In this work, we successfully synthesize CsSn_1–*x*_Ge_*x*_I_3_ thin films by alloying GeI_2_ into CsSnI_3_ through sequential thermal evaporation. We found the Ge^2+^ is well-dispersed in the films on all relevant length scales using scanning electron microscopy with - energy-dispersive X-ray spectroscopy mapping (in-plane micron scale), X-ray photoelectron spectroscopy depth profiling (through-plane nm-scale) and atom probe tomography (3D nm-scale). Ge is oxidized in preference to Sn^2+^ in ambient conditions. This occurs in the top 3 nm of the film and significantly retards the oxidation of Sn^2+^, resulting in superior stability. Above all, the Ge dopant decreases the lattice thermal conductivity of CsSnI_3_ by 21 ± 1%, resulting in an ultralow value of 0.26 ± 0.01 W m^–1^K^–1^ for CsSn_0.9_Ge_0.1_I_3_ at 353 K. Through density functional theory (DFT) calculations, we establish that the large drop in lattice thermal conductivity is due to an increased number of low-frequency phonons upon doping with Ge. This makes the scattering interaction of the acoustic phonons stronger, leading to a smaller phonon group velocity and shorter lifetime. Finally, we performed oxidation-time dependent thermoelectric property measurement for CsSn_1–*x*_Ge_*x*_I_3_ thin films, which confirm the superior stability of CsSn_0.9_Ge_0.1_I_3_ films even by oxidation at elevated temperature. This discovery provides a pathway to reduce the lattice thermal conductivity of halide perovskites and simultaneously improve stability in Sn-based halide perovskite materials.

Our perovskite thin films were deposited by sequential thermal evaporation methods. First, we sequentially deposited SnI_2_ and then CsI by thermal evaporation. To achieve mixed metal CsSn_1–*x*_Ge_*x*_I_3_ perovskite films, GeI_2_ was evaporated on the top surface of the bilayer SnI_2_/CsI films. The films with three layers of precursors were baked at 170 °C to form high-quality mixed halide perovskite CsSn_1–*x*_Ge_*x*_I_3_ thin films (Figure 1a). The CsI, SnI_2,_ and GeI_2_ layers were transparent, yellow, and slightly brown color, respectively, and the colors of unbaked CsSnI_3_ and CsSn_1–*x*_Ge_*x*_I_3_ films were sepia and gray, respectively, changing to mirror-black after annealing (Figure S1). Figure 1(b-d) shows scanning electron microscope (SEM) images of CsSnI_3_, CsSn_0.95_Ge_0.05_I_3_ and CsSn_0.9_Ge_0.1_I_3_ thin films, respectively. All films are highly homogeneous, possess full-coverage, and are pinhole-free with the typical halide perovskite morphology. The films show similar grain size, with the average grain diameter around 700 nm (690 ± 220 nm, 760 ± 240 um and 710 ± 210 um for the *x* = 0, 0.05 and 0.10 films, respectively). While the *x* = 0.10 doped film image (Figure 1d) has a different contrast and texture, this film has significantly lower electrical conductivity than the others (*vide infra*) which can affect the SEM contrast. It is therefore difficult to make further comparisons of film morphology beyond the grain size. The X-ray diffraction (XRD) patterns (Figure 1e) confirm that our pristine CsSnI_3_ films are in the black orthorhombic phase (B-γ) CsSnI_3_ (space group *Pnma*),^13^ with two dominant peaks at 14.48° (101) and 29.15° (202), respectively. After the introduction of germanium, there is a shift of (101) and (202) peaks, to higher 2θ angles by about 0.06° (Figure S2). This implies that the successful doping of germanium causes a shrinkage of the unit cell, as a result of the substitution of the larger Sn(II) by the smaller Ge(II), which was also observed in CsSn_1–*x*_Ge_*x*_I_3_ perovskite nanocrystals^34^ and powders.^35^ The (011) and (022) peaks also become much more prominent in the spectrum for the Ge-doped samples, implying a change in orientation of the crystallites. Energy-dispersive X-ray spectroscopy (EDS) mapping of CsSn_0.9_Ge_0.1_I_3_ films shows a homogeneous distribution of all four elements on length scales down to 100 nm, and segregation near the grain boundaries was not observed (Figure 1f). The atomic composition (Figure S3) of CsSn_0.9_Ge_0.1_I_3_ thin films calculated from EDS shows that the ratio of Ge to Sn is 1:9, verifying that the as-deposited composition is retained upon baking. We further characterized the 3D elemental distribution by atom probe tomography (APT).^36^ We did not observe any elemental segregation or partitioning of Sn and Ge (Figure 1g). In addition, pair distributions of Sn and Ge follow a random pair distribution, indicating the homogeneous distribution of Sn and Ge in the analyzed volume (Figure S4), and confirming that there is no segregation of Ge within crystal domains. From the X-ray photoelectron spectroscopy (XPS) survey (Figure S5a), a growing intensity of the Ge 2p peak is observed with increasing doping ratio. The depth profile of the Ge 2p peak in CsSn_0.9_Ge_0.1_I_3_ thin films (Figure S5b and S5c) demonstrates that the Ge is present throughout the whole film, not just in the top surface. This is in contrast to our previous work on mixed halide CsSnI_3-x_Cl_*x*_ thin films^29^ where the thermally evaporated chloride substitution was only detected in the top few nanometres of the film. We can therefore conclude that Ge is well-dispersed within our films at all length scales and in all dimensions. This observation is in line with our DFT calculations, which showed that the doping formation energy of CsSn_1–*x*_Ge_*x*_I_3_ is negative (Figure S8 and Note S2), indicating that Ge substitutions are stable in the lattice.

**Figure 1:**
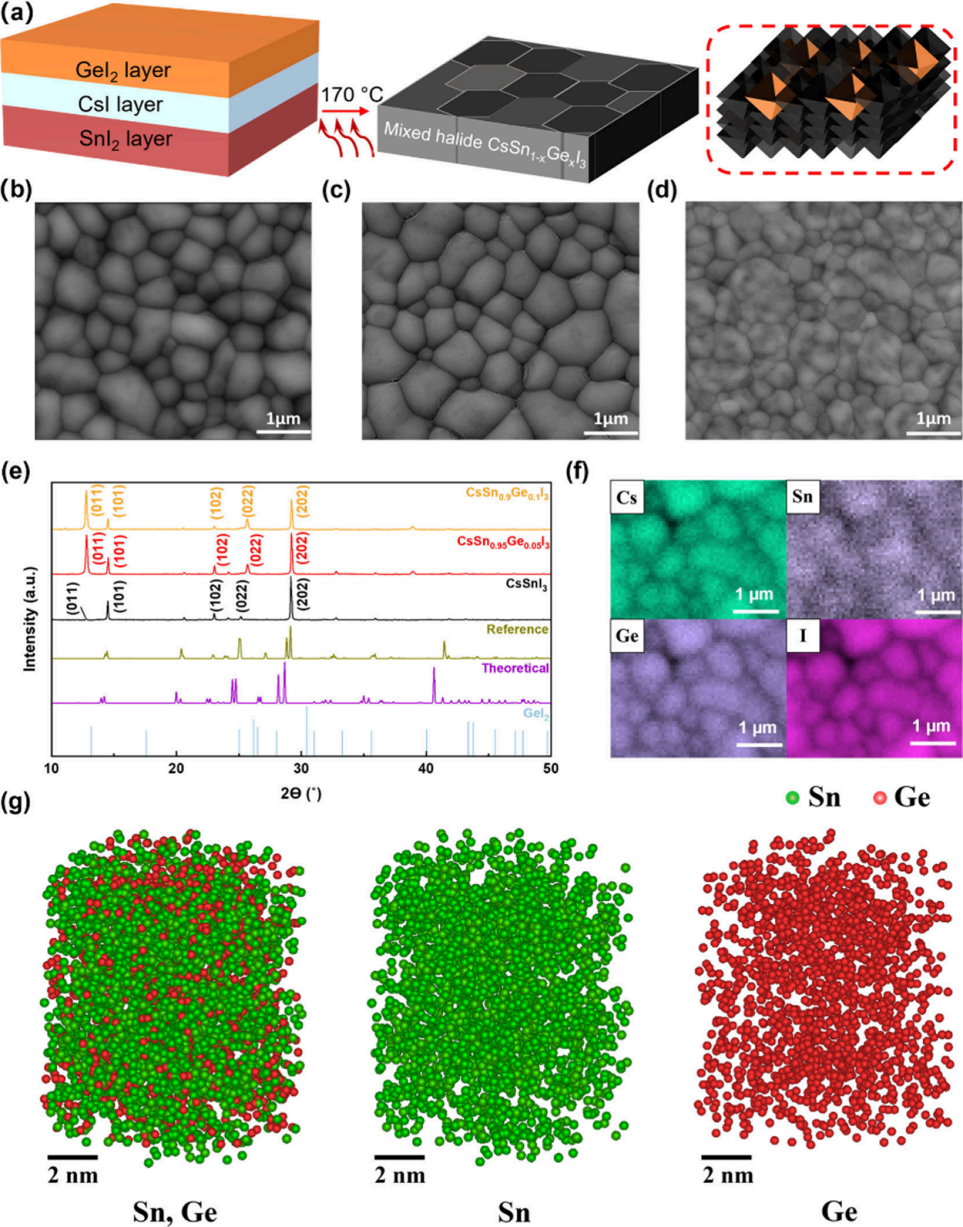
Mixed metal perovskite CsSn_1–*x*_Ge_*x*_I_3_ thin film deposition, morphology, structure and elemental distribution. (a) Schematic of the mixed metal perovskite CsSn_1–*x*_Ge_*x*_I_3_ thin film deposition method. (b-d) Scanning electron microscopy (SEM) images of CsSn_1–*x*_Ge_*x*_I_3_ thin films (*x* = 0, 0.05 and 0.1, respectively). (e) X-ray diffraction (XRD) spectra of CsSn_1–*x*_Ge_*x*_I_3_ (*x* = 0, 0.05 and 0.1) thin films with the reference CsSnI_3_ pattern^13^ and computed CsSnI_3_ and GeI_2_ patterns (from The Materials Project). (f) SEM-EDS element mapping of a CsSn_0.9_Ge_0.1_I_3_ thin film. (g) 3D atom maps of CsSn_0.9_Ge_0.1_I_3_ extracted as a cylinder (radius = 5 nm, height = 10 nm).

Tin-based perovskites are highly air-sensitive due to the oxidation of Sn^2+^ to Sn^4+^, which occurs rapidly because of the low redox potential of Sn^2+^.^28^ Here, we performed three measurements to study the impact of germanium doping on the stability of CsSnI_3_ thin films. First, XRD was used to study the structural stability of the material. Figure 2(a-c) shows the initial XRD patterns of CsSnI_3_, CsSn_0.95_Ge_0.05_I_3_ and CsSn_0.9_Ge_0.1_I_3_. After 6 h exposure in ambient conditions (25 °C, 40% relative humidity (RH)), the black phase CsSnI_3_ film (Figure 2a) experiences a complete phase transition to the yellow orthorhombic phase of CsSnI_3_ (Figure 2d). All peaks of the black phase disappear and one peak of the yellow orthorhombic phase appears at 30.82° (015). Ge-doped CsSnI_3_ thin films are significantly more stable (Figure 2e and f) when exposed to ambient conditions. We quantified the intensity of the two dominant peaks, (101) and (202), before and after 6 h air exposure (Figure S6). After 6 h air exposure, the intensities of the (101) and (202) peaks of CsSn_1–*x*_Ge_*x*_I_3_ thin films decreased in all cases, but the rate of decrease reduced as a function of Ge-doping. The intensity of the (101) and (202) peaks of CsSn_0.9_Ge_0.1_I_3_ thin films reduced to 25.3% and 23.1% of their original values, respectively, which is clearly higher than that of pristine CsSnI_3_ films, retaining only 0% and 1.6% of original intensities.

**Figure 2:**
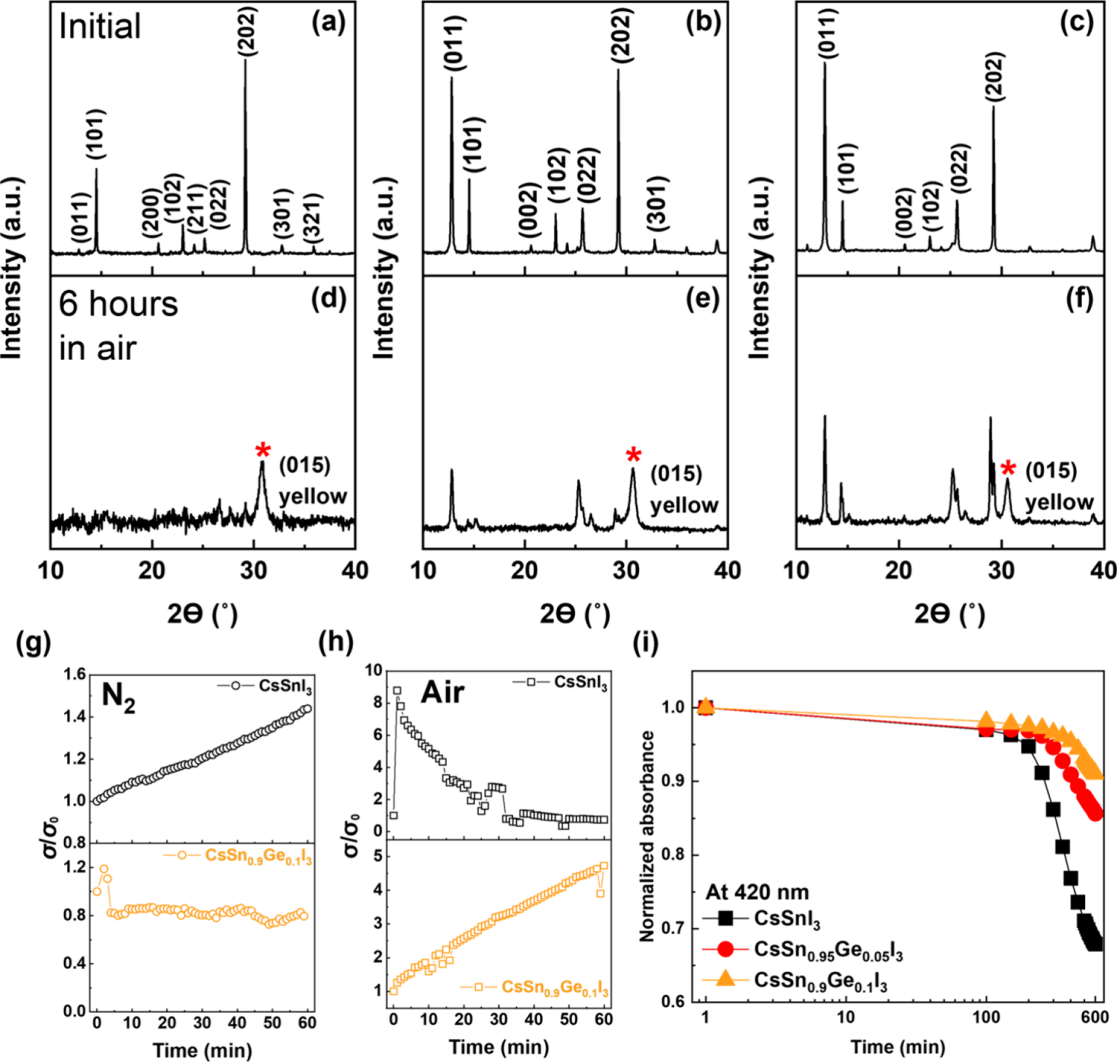
Relative stability of CsSn_1–*x*_Ge_*x*_I_3_ (*x* = 0, 0.05 and 0.1) thin film. XRD patterns of (a) CsSnI_3_, (b) CsSn_0.95_Ge_0.05_I_3_ and (c) CsSn_0.9_Ge_0.1_I_3_ thin films. (d-f) XRD patterns of (d) CsSnI_3_, (e) CsSn_0.95_Ge_0.05_I_3_ and (f) CsSn_0.9_Ge_0.1_I_3_ thin films after 6 h air exposure (25 °C, 40% RH). The red asterisk represents the (015) peak of the yellow orthorhombic phase of CsSnI_3_. Time dependent electrical conductivity of CsSnI_3_ and CsSn_0.9_Ge_0.1_I_3_ thin films in (g) nitrogen atmosphere (H_2_O < 0.1 PPM and O_2_ < 0.1 PPM) and (h) in air (25 °C, 40% RH). (i) Time dependent normalized absorbance (at 420 nm) of CsSnI_3_, CsSn_0.95_Ge_0.05_I_3_ and CsSn_0.9_Ge_0.1_I_3_ thin films in air (25 °C, 40% RH).

Electrical conductivity stability measurements were performed both in a nitrogen filled glovebox (H_2_O < 0.1 PPM and O_2_ < 0.1 PPM) and under ambient conditions (25 °C, 40% RH) for a total of 60 min. The initial conductivities of CsSnI_3_ and CsSn_0.9_Ge_0.1_I_3_ thin films were 1.2 S cm^–1^ and 3.8 S cm^–1^, respectively. When tested in N_2_ (Figure 2g), CsSnI_3_ thin films showed a slight increase of conductivity with time, while in the CsSn_0.9_Ge_0.1_I_3_ thin films the conductivity remained constant. The slow increase in conductivity for CsSnI_3_ is probably due to the spontaneous generation of Sn vacancies in the former case, or a slow oxidation from residual oxygen and water in the glovebox.^37^ In contrast, Ge can occupy these Sn vacancies in the latter case and then improve the stability even in anaerobic environment.^34^ When tested in air (Figure 2h), the conductivity of CsSnI_3_ thin films dramatically increased by a factor of 8.8 in the first minute and then continuously decreased to 73% of the initial value after 1 h of oxidation.^29^ Our previous work has shown that the initial increase then long-term degradation of electrical conductivity of CsSnI_3_ films in air is due to a competition between generation of free carriers caused by oxidation over short time scales and reduction in charge mobility due to increased defect density at longer time scales.^29^ On the other hand, the conductivity of CsSn_0.9_Ge_0.1_I_3_ thin films grows more slowly when exposed to air. After 60 min oxidation, the conductivity increased by a factor of almost 5, but had not yet reached a peak. The presence of Ge in the films is clearly slowing down the oxidative self-doping which could derive from Sn^2+^ to Sn^4+^ or Ge^2+^ to Ge^4+^ oxidation. The Ge-doped CsSnI_3_ thin films also show higher stability of their optical properties (optical absorbance), see Figure S7. After 10 h exposure to ambient conditions, the absorbance at 420 nm (Figure 2i) of CsSnI_3_, CsSn_0.95_Ge_0.05_I_3_ and CsSn_0.9_Ge_0.1_I_3_ reduces to 68%, 86% and 91% of the initial values, respectively. Collectively, these measurements show that Ge doping significantly improves the stability of CsSnI_3_ thin films, which is consistent with previous reports.^34,35,38^

To understand more deeply the origin of enhanced stability in the mixed halide perovskite, we used X-ray photoelectron spectroscopy (XPS) to analyze the tin oxidation states (all samples were exposed to air for 3 min prior to measurement). The presence of Sn^4+^ is clear in the Sn 3d spectra for all three perovskite films (Figure 3(a-c)). Nevertheless, the ratio of fitted Sn^4+^ to Sn^2+^ at the surface progressively decreases with the increase of germanium doping (Figure 3g), something that has previously been reported in FA_0.75_MA_0.25_Sn_1–*x*_Ge_*x*_I_3_ thin films.^39^ This reduction indicates that the germanium content slows the oxidation of Sn^2+^ to Sn^4+^ at the film surface and can explain the improved thin film air stability in the Ge-doped systems. When depth profiling the Ge 2p_2/3_ peak in CsSn_0.9_Ge_0.1_I_3_ films (Figure 3d), it is evident that a Ge^4+^ signal dominates at the surface, but is not present beyond a depth of 3 nm, which suggests that no Ge^2+^ oxidation is occurring beyond 3 nm into the film on this time scale. The Sn MNN Auger electron spectroscopy (AES) spectra of CsSn_1–*x*_Ge_*x*_I_3_ films (Figure 3e and 3f) show a broad line shape with multiple fitted peaks (a, b, c and d), which have been assigned in CsSnI_3–x_Cl_*x*_^29^ and other tin-systems.^32,40−42^ Peak c is related to the presence of Sn^4+^, and for the same oxidation time, Peak c is visible in CsSnI_3_ films to a 6 nm etching depth (Figure 3h), while for CsSn_0.9_Ge_0.1_I_3_ films, it is only present at the surface and is no longer detectable at just 3 nm depth in the film. There appears to be competitive oxidation between Ge^2+^ and Sn^2+^ at the surface of CsSn_0.9_Ge_0.1_I_3_ films. Ge is oxidized preferentially and this slows down or delays Sn oxidation. In CsSn_0.9_Ge_0.1_I_3_ films Sn^4+^ is only detected at the film surface while Ge^4+^ is detectable at 3 nm depth (Figure 3h). A schematic of the Sn oxidation and Ge protection process is exhibited in Figure 4. Furthermore, through DFT calculations, we note that the doping formation energy of Ge-doped CsSnI_3_ is negative (Figure S6 and Note S2), meaning that Sn vacancies are stabilized by Ge dopants.

**Figure 3:**
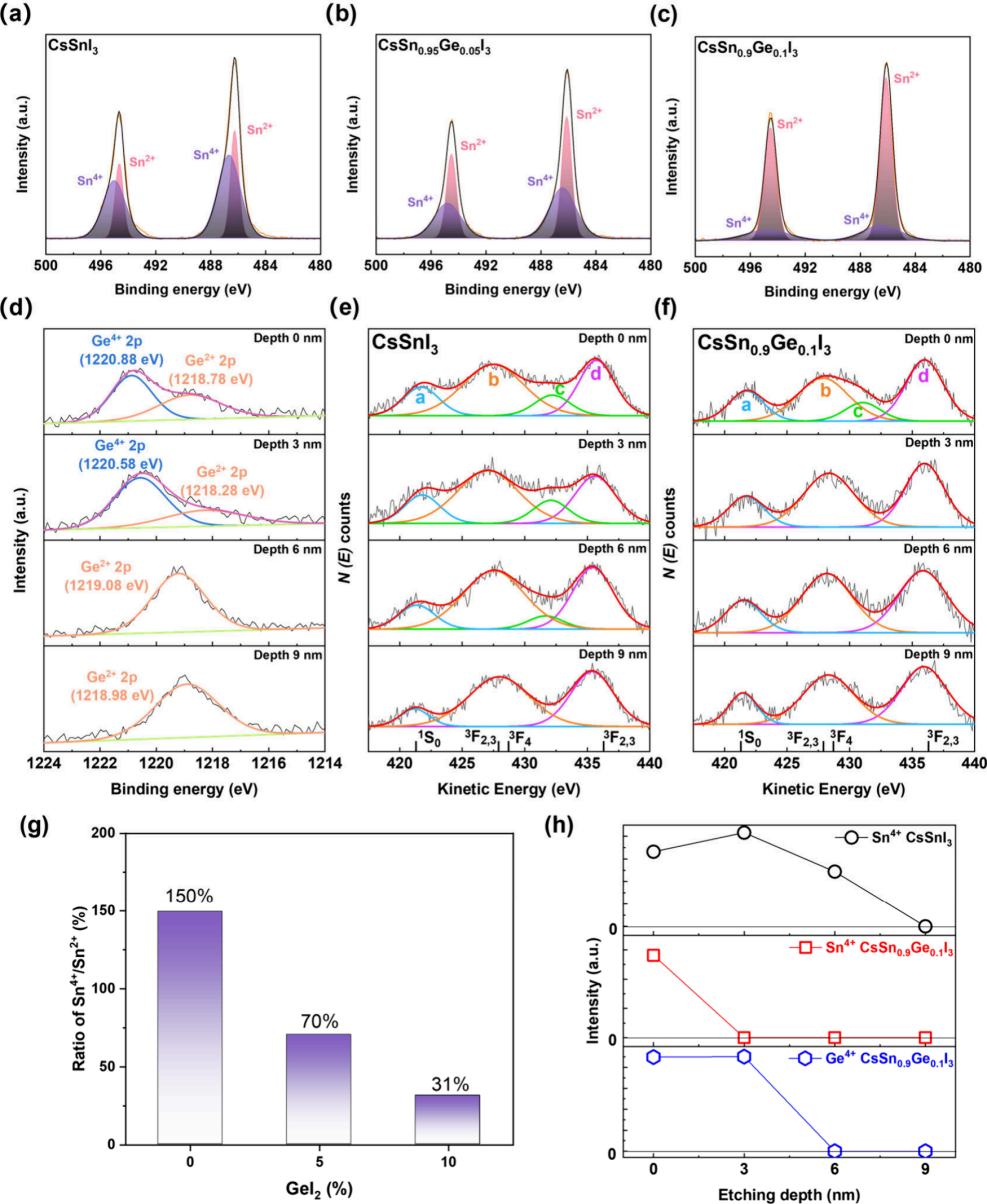
Sn and Ge oxidation states in CsSn_1–*x*_Ge_*x*_I_3_(x = 0 and 0.1) thin film. (a-c) XPS Sn 3d spectra of CsSnI_3_, CsSn_0.95_Ge_0.05_I_3_ and CsSn_0.9_Ge_0.1_I_3_ thin films at top surface. Experimental (orange) and fitted (black) curves are shown along with the fitted peaks (shaded). (d) XPS spectra of Ge 2p in CsSn_0.9_Ge_0.1_I_3_ as a function of etching depth. AES Sn MNN (as a function of etching depth from 0 to 9 nm), (e) CsSnI_3_ and (f) CsSn_0.9_Ge_0.1_I_3_. (g) The ratio of Sn^4+^ to Sn^2+^ at the film surface (depth 0 nm) as a function of GeI_2_ inclusion determined from the fitted Sn 3d_5/2_ spectra (a-c). (h) Photoelectron counts of fitted peak c (related to Sn^4+^) in (e) and (f) and intensity of fitted Ge^4+^ 2p peak in (d) as a function of etching depth.

**Figure 4:**
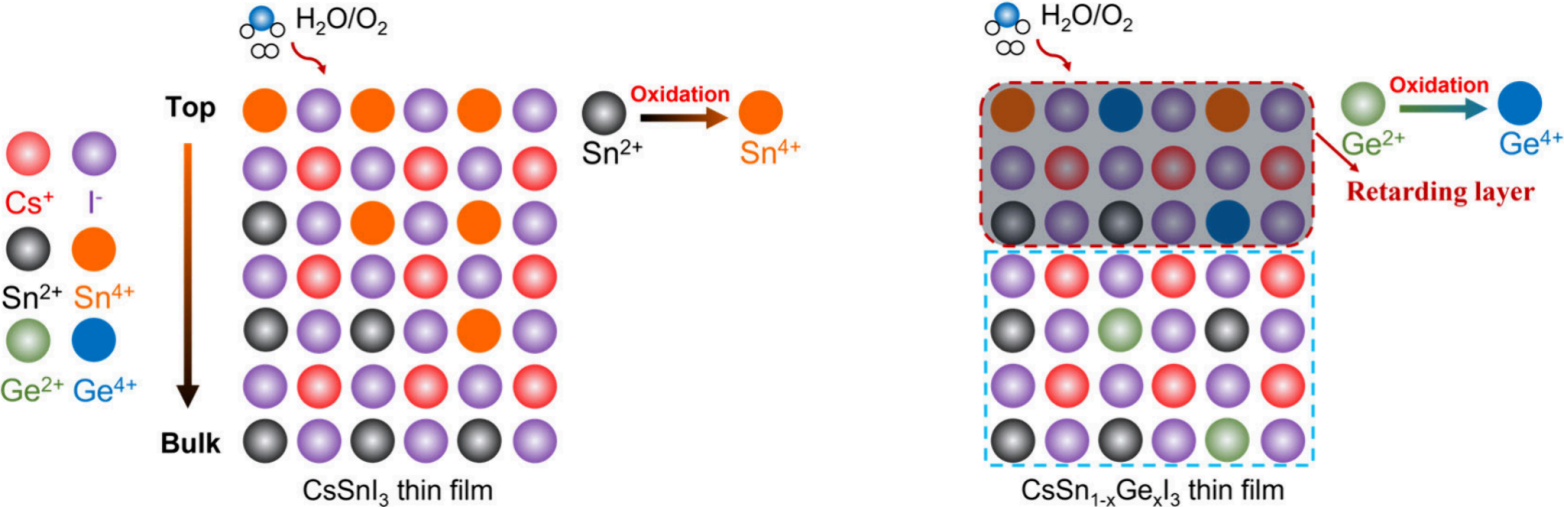
Schematic of Sn and Ge oxidation in CsSn_1–*x*_Ge_*x*_I_3_ thin films.

Figure 5 illustrates the thermoelectric properties of CsSn_1–*x*_Ge_*x*_I_3_ (x = 0, 0.05 and 0.1) perovskite films. As there is an air exposure of about 1 min for loading samples, the initial electrical conductivity of pristine CsSnI_3_ thin film is quite high, 21 ± 8 S cm^–1^ at 293 K (Figure 5a). The initial σ of CsSn_1–*x*_Ge_*x*_I_3_ (*x* > 0) is slightly lower, at 6 ± 4 S cm^–1^ (x = 0.05) and 0.7 ± 0.3 S cm^–1^ (x = 0.1), which is consistent with the suppression of the oxidation of Sn (II) to Sn (IV). All samples exhibit an inverse relationship between electrical conductivity and temperature, indicating a semiconducting transport behavior. A positive Seebeck coefficient is obtained for all three perovskite films (Figure 5b), which indicates that the dominant charge carriers are holes. There is a slightly larger initial Seebeck coefficient in CsSn_1–*x*_Ge_*x*_I_3_ films with increasing Ge content, from 108 ± 5 μV K^–1^ for pristine CsSnI_3_ to 140 ± 26 μV K^–1^ for CsSn_0.9_Ge_0.1_I_3_ at 293 K, which is in agreement with the lower initial electrical conductivity due to a reduced level of Sn (II) to Sn (IV) oxidation. Although, CsSn_0.9_Ge_0.1_I_3_ has a marginally higher *S*, the low σ of CsSn_0.9_Ge_0.1_I_3_ leads to a power factor (PF) (0.02 ± 0.01 μW cm^–1^K^–2^ at 293 K) that is much lower than the PF of undoped CsSnI_3_ (0.20 ± 0.04 μW cm^–1^K^–2^ at 293 K) (Figure S9a).

**Figure 5:**
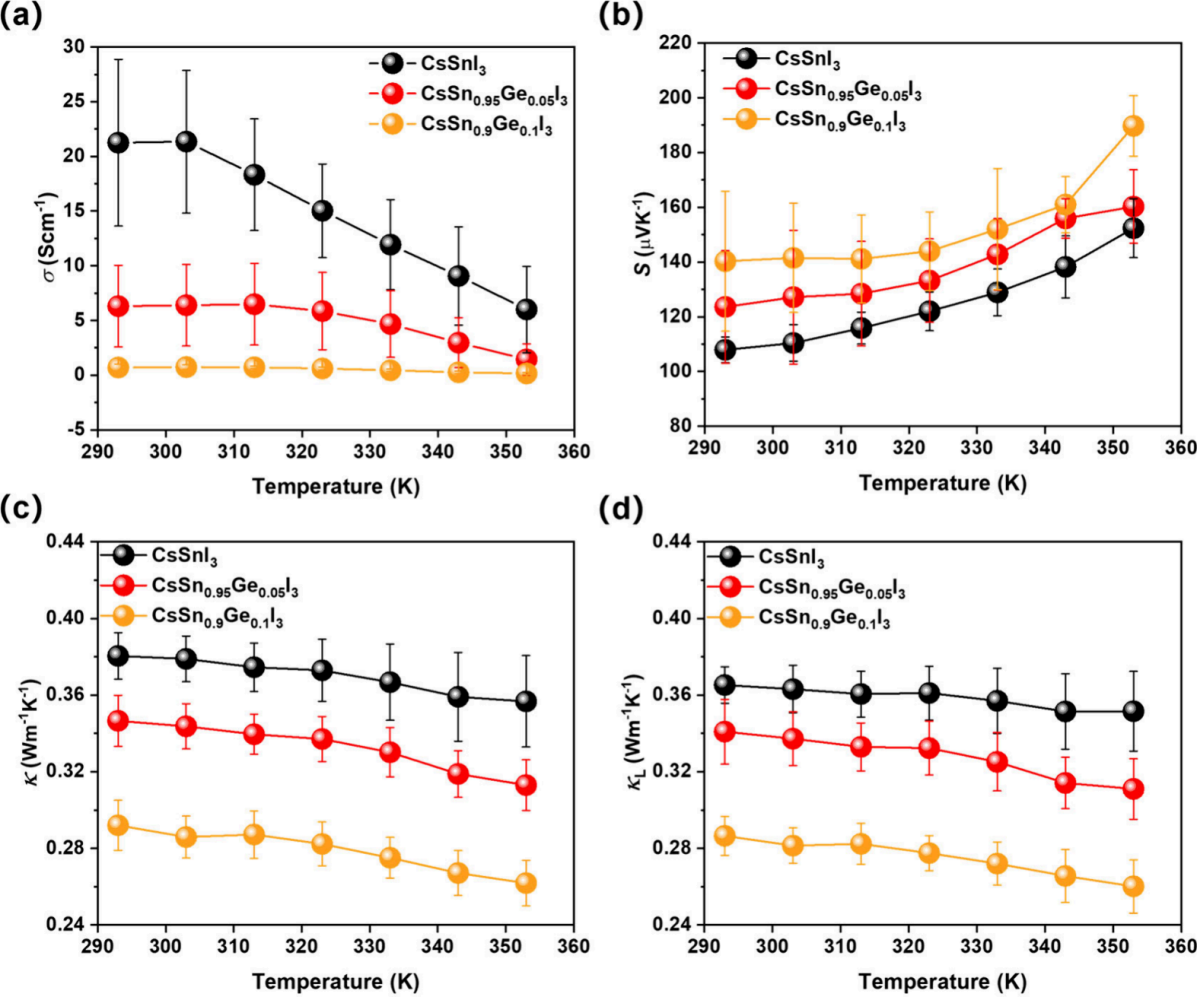
Thermoelectric properties of CsSn_1–*x*_Ge_*x*_I_3_(x = 0, 0.05 and 0.1) thin film. (a) Electrical conductivity. (b) Seebeck coefficients. (c) Total thermal conductivity. (d) Lattice thermal conductivity.

Owing to the rattling mode structure, all-inorganic CsSnI_3_ possesses ultralow thermal conductivity, 0.37 ± 0.01 W m^–1^K^–1^ for our pristine CsSnI_3_ film at 293 K (Figure 5c). We find that Ge substitution can substantially reduce the thermal conductivity, decreasing to 0.34 ± 0.02 W m^–1^K^–1^ for CsSn_0.95_Ge_0.05_I_3_ and 0.29 ± 0.01 W m^–1^K^–1^ for CsSn_0.9_Ge_0.1_I_3_ at 293 K, respectively. Since the electrical conductivity is low, these values approximate to the lattice thermal conductivity (κ_L_). The lattice thermal conductivity of all films, calculated by assuming the Sommerfeld value of the Lorenz number,^29^ decreases as the temperature increases, resulting in the lowest κ_L_ value of 0.26 ± 0.01 W m^–1^K^–1^ for CsSn_0.9_Ge_0.1_I_3_ at 353 K (Figure 5d). The decrease of lattice thermal conductivity in the Ge-doped films compared to the nondoped films (x = 0) could be due to alloy scattering^43^ or an anharmonic cluster rattling mechanism^33^ caused by uneven bond length distribution between Ge–I and Sn–I bond. The decrease is quite significant considering that the thermal conductivity of undoped CsSnI_3_ is already in the ultralow regime, and the thermal conductivity of undoped CsGeI_3_ is expected to be marginally higher (though still low at ∼0.59 W/mK).^44^ The origins of this reduced thermal conductivity are explored below. In their initial state, all of the films have quite low PF and *zT* (Figure S9b) due to their low electrical conductivity, but in section 2.5 we will look at optimization of *zT* through controlled oxidation.

We used DFT to model the lattice thermal conductivity (κ_L_) and phonon properties of CsSn_1–*x*_Ge_*x*_I_3_ perovskites, computing the phonon dispersion relationship, phonon density of states, phonon group velocity and phonon scattering lifetime (Figure 6). Specifically, we modeled CsSn_1–*x*_Ge_*x*_I_3_ with x = 0.0 and 0.125 in the orthorhombic structure (Figure 6a and 6b). By combining the *ab initio* molecular dynamic (AIMD) simulations, the temperature-dependent effective potential (TDEP) method was used to calculate the phonon dispersion as shown in Figure 6c. The phonon dispersion of CsSnI_3_ shows ultralow frequency optical phonon modes near the Γ point, which can induce strong scattering processes between the acoustic and optical phonons. This strong scattering occurs because of the law of conservation of energy and momentum in phonon scattering processes. Phonon scattering therefore happens preferentially between phonon branches closer in energy or momentum.^45^ Under the theoretical framework of phonon Boltzmann transport equation (BTE), the lattice thermal conductivity κ_L_ can be expressed as
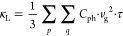
1where *C*_ph_, *v*_g_, and τ are the phonon heat capacity, phonon group velocity, and phonon scattering lifetime.^46,47^ From the square of the phonon group velocity presented in Figure 6f,  is on average smaller in CsSn_0.875_Ge_0.125_I_3_ than in CsSnI_3_ (Figure 6f). This is because CsSn_0.875_Ge_0.125_I_3_ possesses more low-frequency phonons than pure CsSnI_3_, which can be observed in the phonon density of states in Figure 6e in the region below 1 THz.

**Figure 6:**
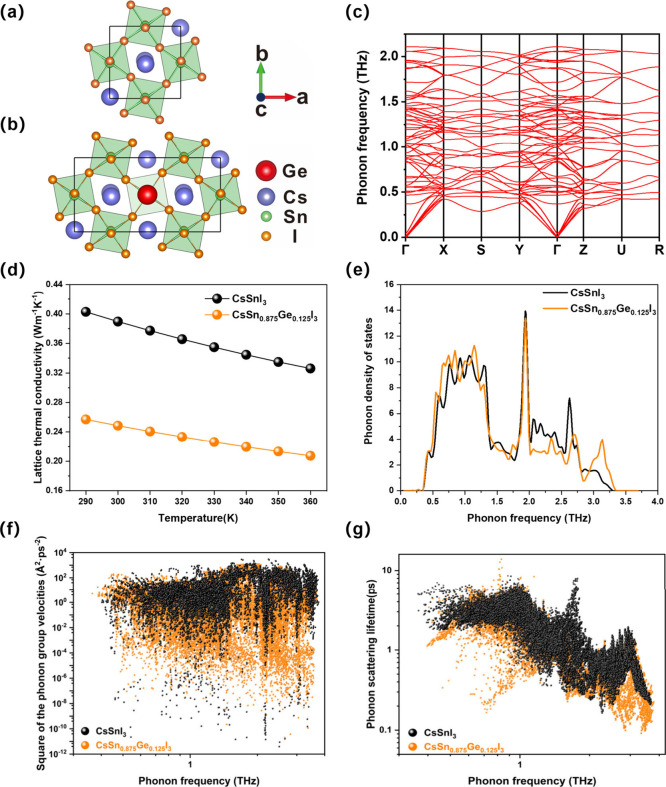
Calculated phonon properties and lattice thermal conductivities of CsSn_1–*x*_Ge_*x*_I_3_(x = 0, 0.125) from DFT simulations. (a) and (b) DFT simulation cells for x = 0 and x = 0.125 respectively. (c) Calculated phonon dispersion of CsSnI_3_. (d) Calculated lattice thermal conductivities. (e) Calculated phonon density of states. (f) Square of the calculated phonon group velocities. (g) Calculated phonon scattering lifetimes.

Simultaneously, the lower frequency phonons in CsSn_0.875_Ge_0.125_I_3_ will bring strong three-phonon scattering because of the larger phonon scattering phase space. The “phase space” is the fraction of three-phonon scattering processes that are allowed by conservation of energy, which can be expressed as^48^

where *p*, *p*′, *p*″s indicate the phonon branches. *q*, *q*′, ***Q*** represent points in momentum space, and ω is the frequency of a specific phonon. BZ is the Brillouin zone. The quantity, Ω = *n*_*p*_^3^*V*_*BZ*_^2^, is a normalization factor equal to the unrestricted phase space for each type of process. *n*_*p*_ is the number of phonon branches whose Brillouin zone has volume *V*_*BZ*_. δ[*ω*_*p*_(***q***) + ω_*p*′_(***q***′)−ω_*p*″_(***q + q***′ ***–Q***)] describes the conservation of energy and it is the reason why the three-phonon scattering more easily happens among close phonon branches. From the phonon dispersion of CsSnI_3_ (Figure 6c), it can be seen that the optical phonon mode is very close to three acoustic modes around the Γ point.

The stronger phonon scattering process results in a shorter phonon lifetime as shown in Figure 6g. Because we adopt the same mesh-grids for sampling in q-space integral of *κ*_*L*_, the volume of orange points from CsSn_0.875_Ge_0.125_I_3_ is smaller the black points from pure CsSnI_3_ in Figure 6g, especially below 1THz and near 3THz. Hence, the smaller phonon group velocity and smaller phonon lifetime in CsSn_0.875_Ge_0.125_I_3_ drive the reduction of *κ*_*L*_ in accordance with Equation 1. Our simulated results of *κ*_*L*_ (Figure 6d) exhibit remarkable agreement with experimental measurement. Our first-principles calculations demonstrate that the *κ*_*L*_ can be reduced by 36% from 0.390 to 0.249 W m^–1^K^–1^ through doping 12.5% Ge in CsSnI_3_ at room temperature (300 K), which compares well to the 21% reduction observed experimentally for 10% Ge-doping.

Due to the increase of electrical conductivity resulting from the short air exposure, we explored the oxidation time dependence of the thermoelectric properties of CsSnI_3_ (Figure S10), CsSn_0.95_Ge_0.05_I_3_ (Figure S11) and CsSn_0.9_Ge_0.1_I_3_ (Figure S12) thin films (at room temperature), respectively. All films showed a slight increase of electrical conductivity and a mild decrease of Seebeck coefficient after in situ air exposure, both consistent with an increase in charge carrier density caused by the oxidation of Sn^2+^ to Sn^4+^. The room-temperature thermal conductivity of undoped CsSnI_3_ increases from 0.40 W m^–1^K^–1^ to the largest value of 0.41 W m^–1^K^–1^ after 15 min oxidation consistent with an increase in electronic thermal conductivity. The thermal conductivity of *x* > 0 CsSn_1–*x*_Ge_*x*_I_3_ is lower than CsSnI_3_ at all oxidation times. The pristine CsSnI_3_ thin films show a peak and then slight reduction of thermoelectric performance with room temperature oxidation time (Figure S13) due to mild film degradation. On the other hand, the Ge-doped (*x* > 0) CsSn_1–*x*_Ge_*x*_I_3_ films show no such decrease and *zT* actually increases over the same period of time due to the oxidative self-doping process. When performing an accelerated oxidation time dependence of the thermoelectric properties of CsSnI_3_ (Figure S14), CsSn_0.95_Ge_0.05_I_3_ (Figure S15) and CsSn_0.9_Ge_0.1_I_3_ (Figure S16) thin films at higher temperature (353 K), a similar pattern is observed, with Ge-doped films showing enhanced stability in all thermoelectric properties (Note S3).

In summary, we have demonstrated a simple sequential thermal vapor deposition method to form high quality all-inorganic Sn–Ge perovskite films from its precursor materials. Our films show pinhole free and homogeneous morphology. Ge and Sn atoms evenly distribute though the whole film and segregation is not observed at any length scale from the SEM-EDS mapping (in-plane micron scale), XPS depth profiling (through-plane nm-scale) and APT (3D nm-scale). Moreover, by alloying GeI_2_ into CsSnI_3_ films, the optical, electrical and structural stability show a significant improvement. Through depth profiling by XPS and AES, we found that the origin of the improved stability in all-inorganic halide perovskites CsSn_1–*x*_Ge_*x*_I_3_ films is due to the oxidation priority of Ge^2+^. As Ge^2+^ is oxidized in preference to Sn^2+^, the oxidized products become a native shield, which protects the underlying Sn^2+^ against further oxidation, slowing down degradation processes. Additionally, we found that Ge inclusions decrease the lattice thermal conductivity, leading to an ultralow lattice thermal conductivity of 0.26 ± 0.01 W m^–1^K^–1^ for CsSn_0.9_Ge_0.1_I_3_ at 353 K. Through DFT calculation, the origin of the significant reduction of lattice thermal conductivity was found to be an increased number of low-frequency phonons in Ge-doped systems that lead to stronger scattering between the acoustic phonons. Finally, our oxidation-time dependent thermoelectric property measurements demonstrate that the CsSn_0.9_Ge_0.1_I_3_ films present improved thermoelectric stability. The ability to stabilize tin perovskites by Ge-doping is important for their development in optoelectronic devices, but in the area of thermoelectrics, where the tin-based materials have the highest reported figure of merit among the halide perovskites, the simultaneous decrease in thermal conductivity is an additional huge advantage. Despite our success in reducing the thermal conductivity and enhancing the stability of tin-based perovskites, *zT* at optimal oxidation level decreased slightly with increasing doping (Figures S17–S19). It may be that high levels of Ge-incorporation in the lattice increase the lattice disorder and decrease the Seebeck coefficient. There is, therefore, still a trade-off in stability versus performance. Further investigation into this trade-off and approaches to minimize it, such as by exploring different B-site dopants of different size or dopants on other sites should be explored, along with their effect on lattice disorder and stability.

## Methods

### Thermal Evaporation of CsSn_1–*x*_Ge_*x*_I_3_ Thin Films

A sequential thermal evaporation method was used to deposit CsSn_1–*x*_Ge_*x*_I_3_ thin films. For pristine CsSnI_3_ thin films, tin(II) iodide (SnI_2_, 99.99%, Sigma-Aldrich) was first thermally evaporated at 2 Å s^–1^ (180 °C), followed by cesium iodide (CsI, 99.9%, Sigma-Aldrich)) at 6 Å s^–1^ (410 °C). For mixed CsSn_1–*x*_Ge_*x*_I_3_ thin films, germanium(II) iodide (GeI_2_, 99.99%, VWR International Ltd.) was evaporated at 1 Å s^–1^ (150 °C) on top of pristine CsSnI_3_ thin films (without any intermediate annealing step) after breaking vacuum to N_2_ atmosphere and changing precursor crucible. For pristine CsSnI_3_ films, the thicknesses of the SnI_2_ and CsI layers were 120 ± 10 nm and 140 ± 10 nm, respectively. For CsSn_0.95_Ge_0.05_I_3_ film, the thicknesses of SnI_2_, CsI and GeI_2_ layers were 110 ± 10 nm, 140 ± 10 nm and 10 ± 5 nm, respectively. For CsSn_0.9_Ge_0.1_I_3_ films, the thicknesses of SnI_2_, CsI and GeI_2_ layer were 100 ± 10 nm, 140 ± 10 nm and 20 ± 5 nm, respectively. The whole thermal evaporation process was performed at 10^–7^ mbar in a dark environment. After thermal evaporation, the as-evaporated thin films were removed from the vacuum chamber and directly baked at 170 °C on a hot plate in a nitrogen filled glovebox without any air exposure. The thickness of all as-synthesized films was 250–300 nm. Figure S21 illustrates the whole film-deposition process.

### Powder X-ray Diffraction

XRD measurements were performed on CsSn_1–*x*_GexI_3_ thin films (on quartz coated glass substrate) using a D5000 X-ray Diffractometer (Siemens) with a Cu–K_α_ source (λ = 1.54 Å).

### Scanning Electron Microscopy

SEM measurements were performed on CsSn_1–*x*_Ge_*x*_I_3_ thin films (on quartz coated glass substrates) using a field-emission scanning electron microscope (FEI Inspect-F). Energy dispersive X-ray spectroscopy (EDS) maps and spectra were taken at 10 kV using an Octane EDS system (EDAX). Multivariate statistical analysis^49^ was performed on the spectrum imaging data set to identify spatial variation in stoichiometry, which confirmed a homogeneous distribution of Ge with respect to Sn.

### Atom Probe Tomography

Atom probe tomography (APT) tips were prepared with a dual-beam focused ion beam (FIB)–scanning electron microscopy (SEM) system (Thermo Fisher Scientific Scios 2 Dual Beam). To protect CsSn_1–*x*_Ge_*x*_I_3_ from damage by the Ga ion beam, we deposited a few hundred nanometre thick carbon layer using a marker prior to deposition of a Pt protective layer.^50^ Except for deposition of the protective layer, we prepared the APT specimens by using a standard FIB lift-out procedure described elsewhere.^51^ All preparation details are illustrated in Figure S22. APT analysis was carried out using a local electrode atom probe (Cameca LEAP 5000 XS) in pulsed laser mode at 30 K. The laser pulse energy and frequency were 60 pJ and 125 kHz, respectively. Data reconstruction and analyses were done with the IVAS 3.8.8 software provided by Cameca Instruments. Movies showing the atom distribution from all angles around the *z*-axis are available online as supplementary files.

### Ultraviolet–visible Spectroscopy

UV–vis absorption spectroscopy was performed on CsSn_1–*x*_Ge_*x*_I_3_ thin films (on quartz coated glass substrate) using a Shimadzu UV-2600 spectrophotometer with a single beam. The baseline was measured with the same quartz coated glass substrate used for thin film deposition. To study UV–vis absorption air stability, the measurement was run every 10 min for total 10 h.

### X-ray Photoelectron Spectroscopy

XPS measurements were performed on CsSn_1–*x*_Ge_*x*_I_3_ thin films (on quartz coated glass substrates) using a Thermo Scientific Nexsa Surface Analysis System with a monochromatic Al K_α_ source under high vacuum (<10^–8^ mbar). The XPS spectra were collected using an X-ray spot size of 100 × 100 μm^2^. To perform depth profile measurements, in situ 2 keV Ar^+^ sputtering 3 s per time was used to etch away the perovskite thin film. The etching depth was calculated from the total etching time corresponding to the total etching thickness (from the film surface to the quartz coated glass substrate). All XPS data were recorded and processed using the Thermo Avantage software.

### Electrical Conductivity Measurement

Time-dependent electrical conductivity measurements were performed by a linear 4-point probe method on CsSn_1–*x*_Ge_*x*_I_3_ thin films (on 15 × 20 mm quartz coated glass substrates) using a Keithley 2400 SourceMeter connected with an Ossila Four-Point Probe System (probe separation 1.27 mm). The perovskite thin films were measured either inside a nitrogen filled glovebox (H_2_O < 0.1 PPM and O_2_ < 0.1 PPM) or in air (25 °C, ∼ 40% RH) every minute for 1 h.

### Thermoelectric Property Measurement

Thermoelectric property measurement was performed on CsSn_1–*x*_Ge_*x*_I_3_ thin films (on measurement chips) using a Linseis Thin Film Analyzer (TFA). A detailed description of this is provided in the Supporting Information (Figure S23 and Note S1). The electronic contribution to thermal conductivity was estimated using the Wiedemann–Franz law (*κ*_*electronic*_*= σLT*), where *L* is the Lorenz number. Our previous work showed that the Lorenz number takes the Sommerfeld value in CsSnI_3_.^29^

### Simulation

All the simulations were based on the density functional theory (DFT) method and implemented in the Vienna ab initio simulation package (VASP).^52^ The projector augmented wave method (PAW)^53,54^ and generalized gradient approximation (GGA) with the Perdew–Burke–Ernzerhof (PBE) exchange-correlation functional were adopted.^55^ The plane-wave cutoff was set to 500 eV and the energy criterion for self-consistent convergence was 1 × 10^–6^ eV. A 4 × 4 × 4 Monkhorst–Pack k-mesh including the Γ point was used to sample the whole Brillouin zone (BZ). The Hellmann–Feynman forces tolerance value was taken as 1 × 10^–4^ eV/Å for all geometrical optimizations. The convergence of the kinetic energy cutoff and k-mesh were examined.

### Phonon Dispersion Simulation

The temperature-dependent effective potential (TDEP) method^56,57^ combined with *ab initio* molecular dynamics (AIMD) simulation has been used to study the phonon properties beyond the quasiharmonic approximation. The supercell adopted included 160 atoms for the AIMD simulation. Born–Oppenheimer AIMD simulations were performed in a canonical ensemble with a fixed number of atoms (N), volume (V) and temperature (T) (NVT) by using the Nosé–Hoover thermostat as implemented in VASP.^58,59^ The time step was set to 1 fs and the total simulation time was 10 ps. The BZ sampling was limited to the Γ point for the AIMD simulations. The static phonon dispersion relations were calculated via the finite-displacement method using the default displacement distance of 0.01 Å by the PHONOPY^60^ code package based on the DFT static calculations in VASP. The 2 × 2 × 2 supercell was adopted for the harmonic force constant calculations. The phonon dispersion relationship and phonon density of states were presented in Figure 6.

### Phonon Properties from Boltzmann Transport Equation (BTE)

based on VASP static calculations, the anharmonic force constants were calculated with the finite-displacement method by thirdorder.py.^45^ With the harmonic (2nd order) and anharmonic (3rd order) interatomic force constants (IFCs), through the program ShengBTE,^45^ the lattice thermal conductivities were calculated by iteratively solving the BTE. The same supercell and k-mesh were used in the DFT simulations to obtain the third-order IFCs, and the interactions between atoms were considered up to the fourth nearest neighbors. The 10 × 10 × 10 q-grids were adopted for the *κ*_*L*_ integration and the convergence of the q-grid mesh size were checked.

The three phonon scattering processes were calculated and the related phonon properties are shown in Figure 6 including the phonon group velocity and phonon scattering lifetime.

## Data Availability

The data that support the findings of this study are available from the corresponding authors upon reasonable request.

## Supporting Information Available

Optical images of perovskite films; analysis of (101) and (202) XRD peaks; SEM-EDS elemental mapping; Nearest neighbor distribution of mixed halide perovskite CsSn1-xGexI3 obtained from atom probe tomography data; XPS depth profiles; absorption spectra of films during oxidation; calculations of doping formation energies; thermoelectric properties as a function of temperature and oxidation; schematic of the deposition process; schematic and images of the atom probe tomography tip formation; details of thermoelectric measurement methodology (PDF)
Supporting video. Distribution of Sn and Ge atoms measured by atom probe tomography (AVI)
Supporting video. Distribution of Sn atoms measured by atom probe tomography (AVI)
Supporting video. Distribution of Ge atoms measured by atom probe tomography (AVI)

## Supplementary Material

jz4c02618_si_001.pdf

jz4c02618_si_002.avi

jz4c02618_si_003.avi

jz4c02618_si_004.avi
