# Supplementary material for: Reduced Thermal Conductivity and Improved Stability by B-Site Doping in Tin Halide Perovskites
Source: J Phys Chem Lett. 2025 Jan 6;16(2):525–36. doi: 10.1021/acs.jpclett.4c02618 (PMC11744799; doi:10.1021/acs.jpclett.4c02618)
Supplement: Supplementary file 1 — jz4c02618_si_001.pdf [file jz4c02618_si_001.pdf]

Supporting Information

Reduced thermal conductivity and improved stability by B-site doping in tin halide perovskites

Weidong Tang, Siyuan Zhang, Tianjun Liu, Chanwon Jung, Se-Ho Kim, Christina Scheu, Shengying Yue, Oliver Fenwick**

Weidong Tang, Tianjun Liu, Oliver Fenwick
School of Engineering and Materials Science, Queen Mary University of London, Mile End Road, London E1 4NS, UK.
E-mail: o.fenwick@qmul.ac.uk

Siyuan Zhang, Chanwon Jung, Se-Ho Kim, Christina Scheu
Max-Planck-Institut für Eisenforschung, Max-Planck-Str. 1, 40237 Düsseldorf, Germany

Se-Ho Kim (Current affiliation)
Department of Materials Science and Engineering, Korea University, Seoul 02841, Republic of Korea.

Shengying Yue
State Key Laboratory for Strength and Vibration of Mechanical Structures, School of Aerospace, Xi'an Jiaotong University, Xi'an 710049, China
E-mail: syyue@xjtu.edu.cn

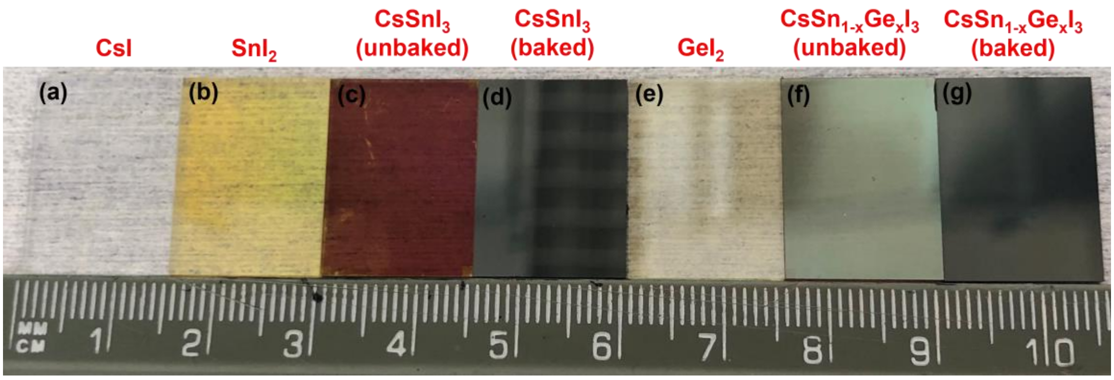

Figure S1. Optical images of precursor and perovskite layers. (a-g) CsI layer, SnI_2 layer, $CsSnI_3$ (unbaked), $CsSnI_3$ (baked), GeI_2 layer, $CsSn_{1-x}Ge_xI_3$ (unbaked), and $CsSn_{1-x}Ge_xI_3$ (baked), respectively.

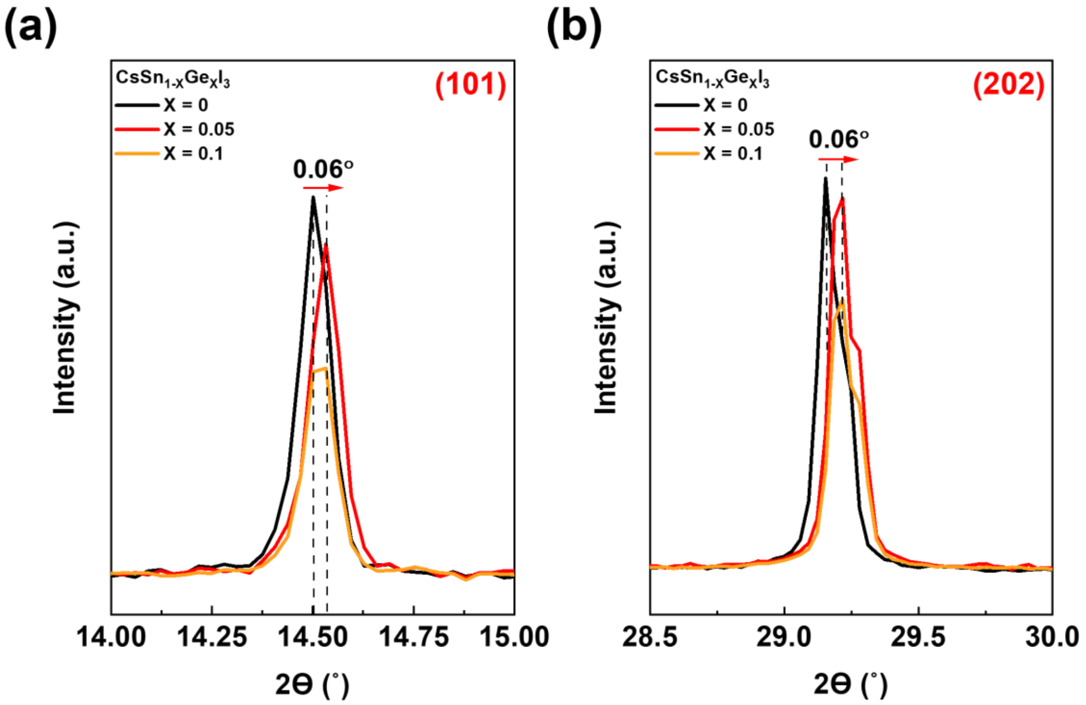

Figure S2. XRD pattern of the dominant peaks of $CsSn_{1-x}Ge_xI_3$ (x = 0, 0.05 and 0.1) thin films. (a) (101) peak and **(b)** (202) peak.

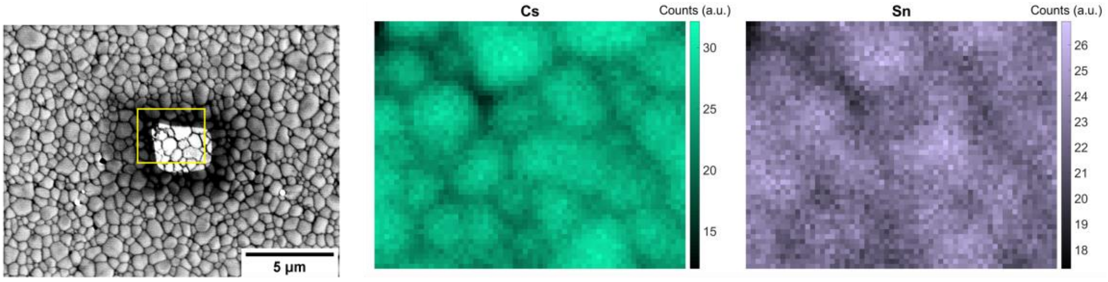

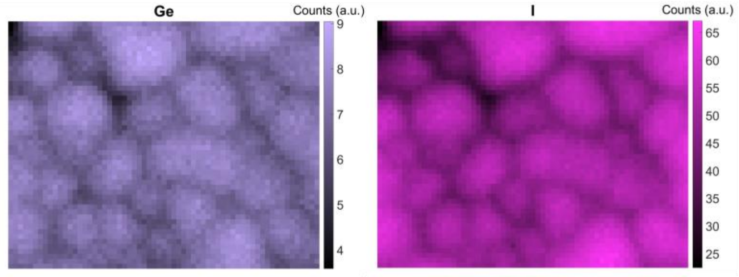

Elements	Atomic %
Ge	2.2
Sn	24.8
I	55.5
Cs	17.5

Figure S3. SEM-EDS elemental mapping and atomic composition of $CsSn_{0.9}Ge_{0.1}I_3$ thin films. The Cs, Sn, Ge and I maps are taken from the region marked with a yellow rectangular box in the SEM image. The atomic % were calculated using the standard Cliff-Lorimer equation.

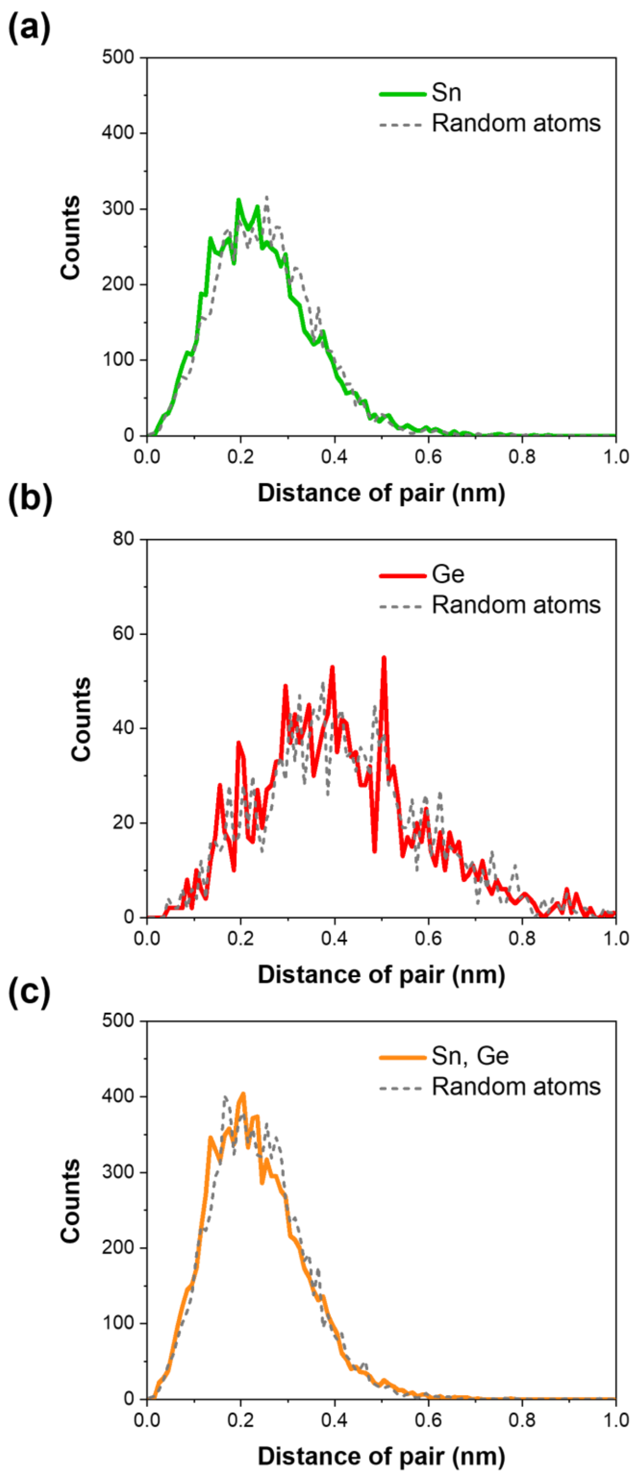

Figure S4. Nearest neighbor distribution of mixed halide perovskite $CsSn_{1-x}Ge_xI_3$ obtained from atom probe tomography data. **(a)** Sn-to-Sn nearest neighbour distance; **(b)** Ge-to-Ge nearest neighbour distance; and **(c)** Sn-to-Ge nearest neighbour distance.

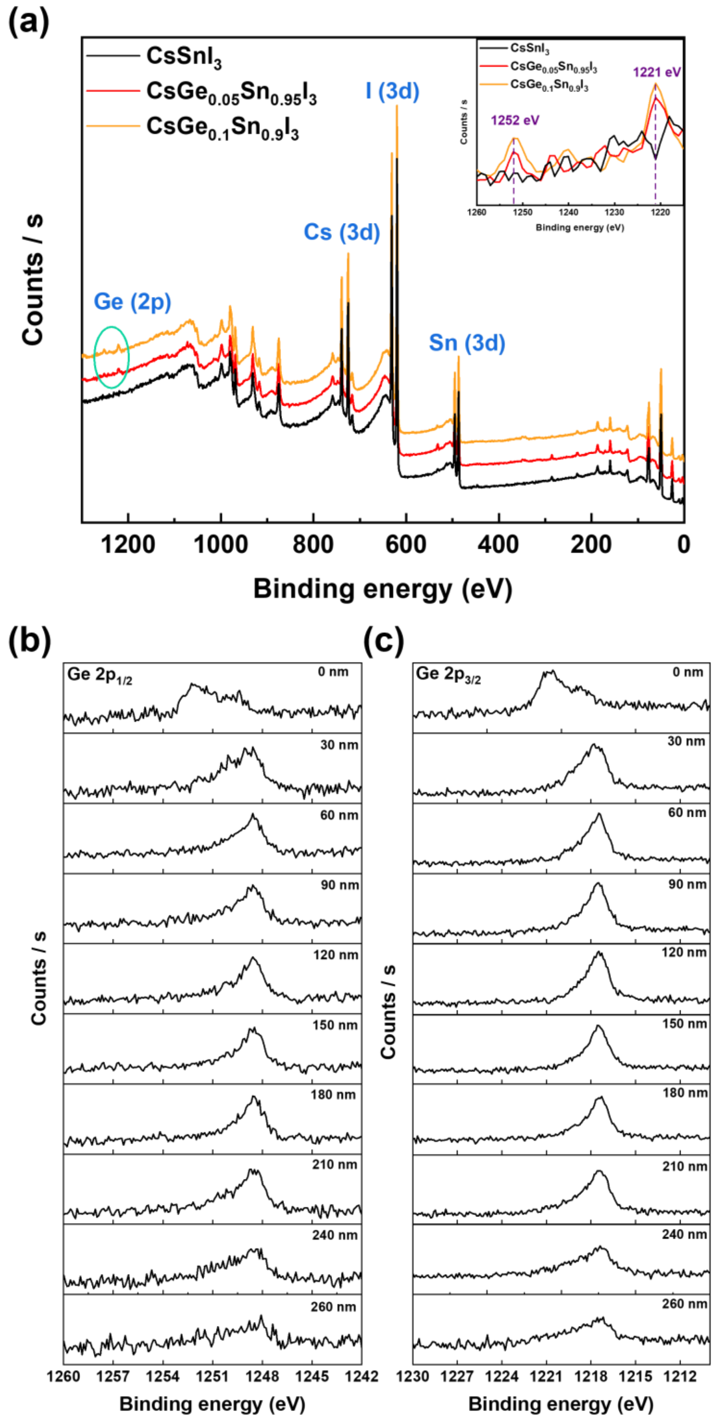

Figure S5. XPS of mixed halide perovskite $CsSn_{1-x}Ge_xI_3$ (x = 0, 0.05 and 0.1) thin films. (**a**) XPS survey of $CsSnI_3$ (black curve), $CsSn_{0.95}Ge_{0.05}I_3$ (red curve) and $CsSn_{0.9}Ge_{0.1}I_3$ (orange curve) thin film, respectively. The inset shows XPS analysis of Ge 2p peak. Depth profile of Ge(2p) peak of $CsSn_{0.9}Ge_{0.1}I_3$ films (**b**) $Ge2p_{1/2}$ and (**c**) $Ge2p_{3/2}$.

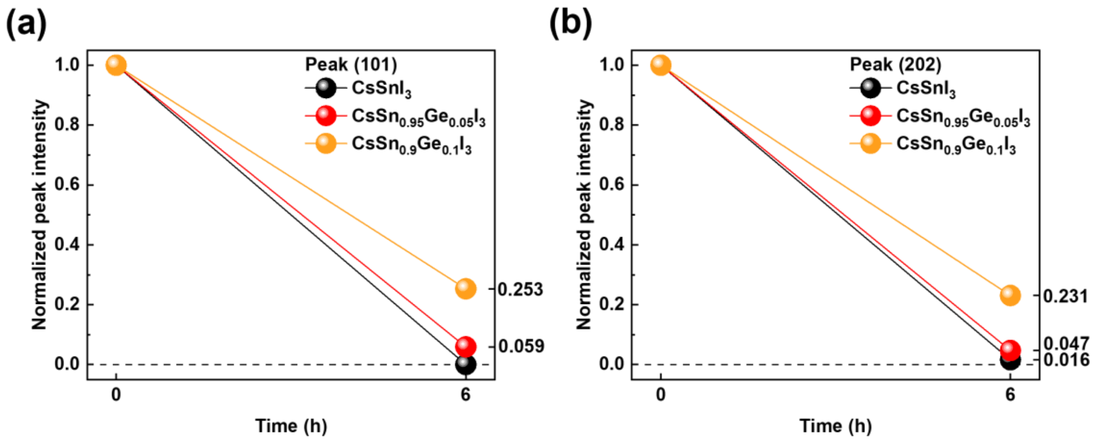

Figure S6. Normalized XRD peak intensity of $CsSn_{1-x}Ge_xI_3$ (x = 0, 0.05 and 0.1) thin films. Peak intensity as a function of air exposure at room temperature for **(a)** the (101) peak; and **(b)** the (202) peak.

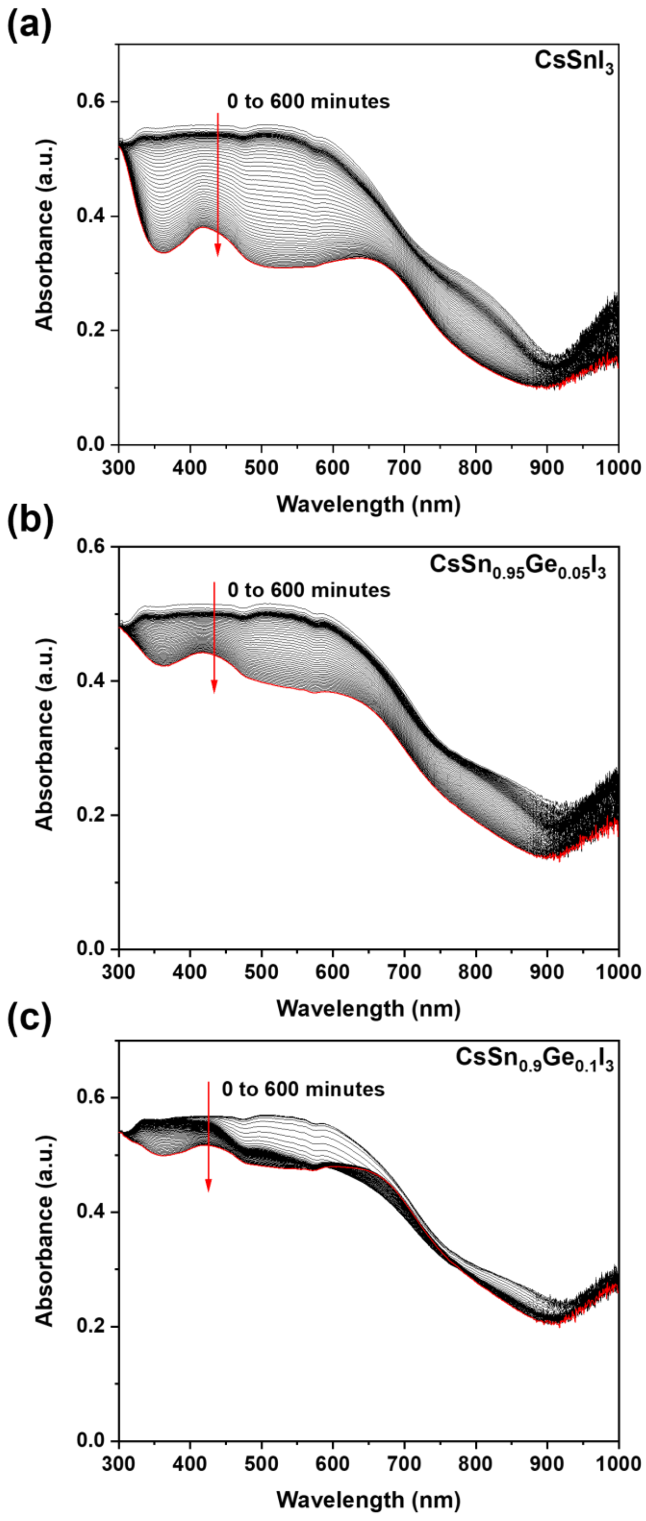

Figure S7. Time dependent UV-vis absorption spectra (from 0 to 600 minutes) of $CsSn_{1-x}Ge_xI_3$ (x = 0, 0.05 and 0.1) thin film in air (25 ˚C, 40% RH). (**a**) $CsSnI_3$, (**b**) $CsSn_{0.95}Ge_{0.05}I_3$ and (**c**) $CsSn_{0.9}Ge_{0.1}I_3$.

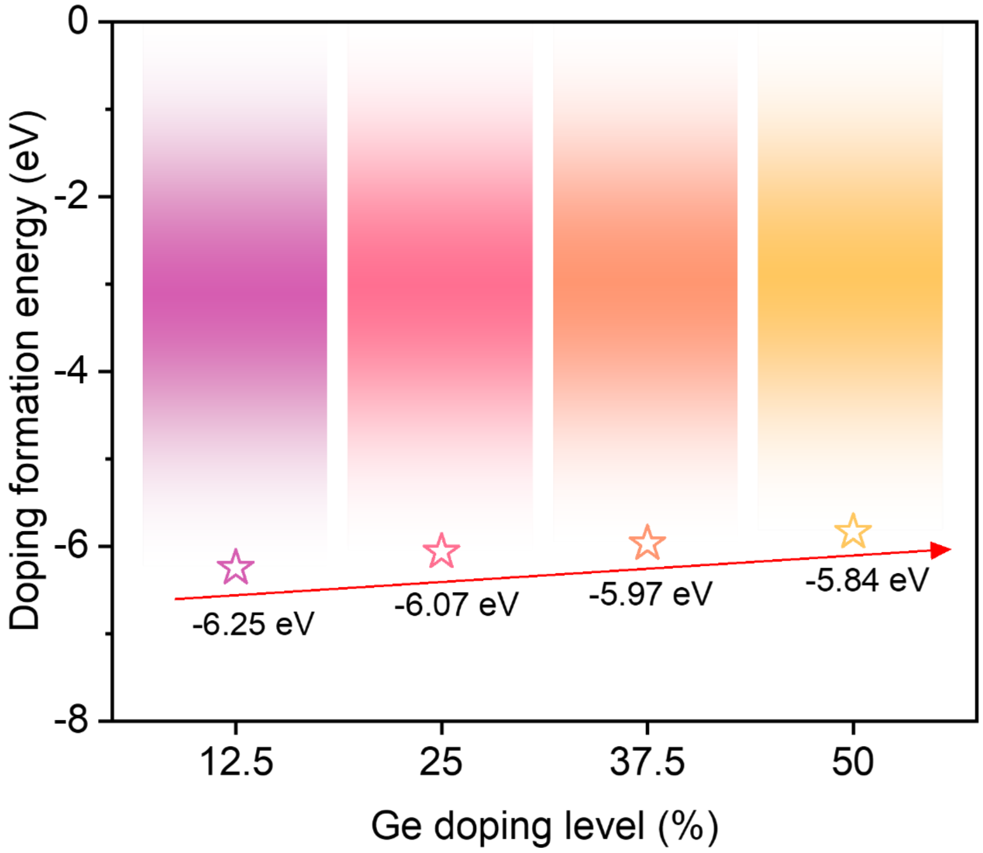

Figure S8. Doping formation energy of $CsSn_{1-x}Ge_xI_3$ (x=0.125, 0.25, 0.375 and 0.5) from the DFT calculations. The energy is slightly increasing with increasing Ge doping level.

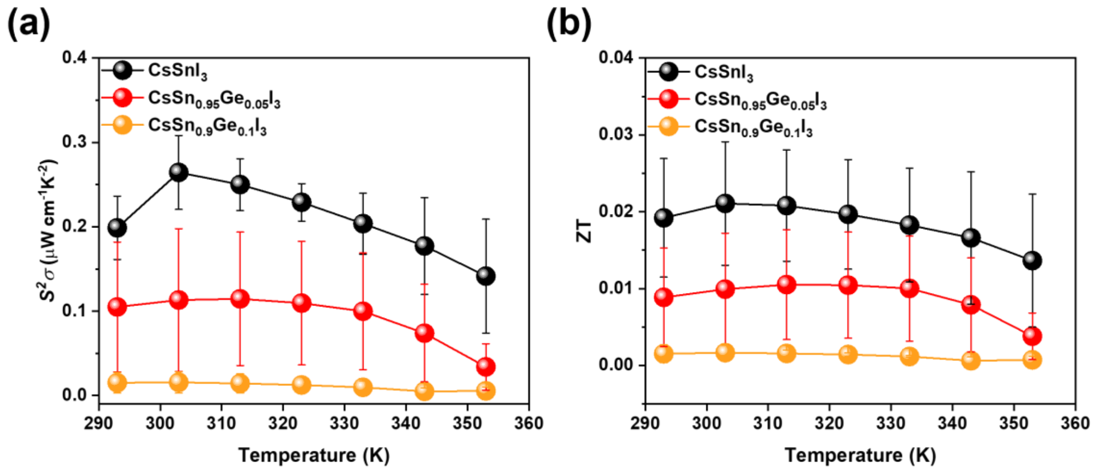

Figure S9. Thermoelectric properties of $CsSn_{1-x}Ge_xI_3$ (x = 0, 0.05 and 0.1) thin film.

(a) Power factor. **(b)** Figure of merit *zT*.

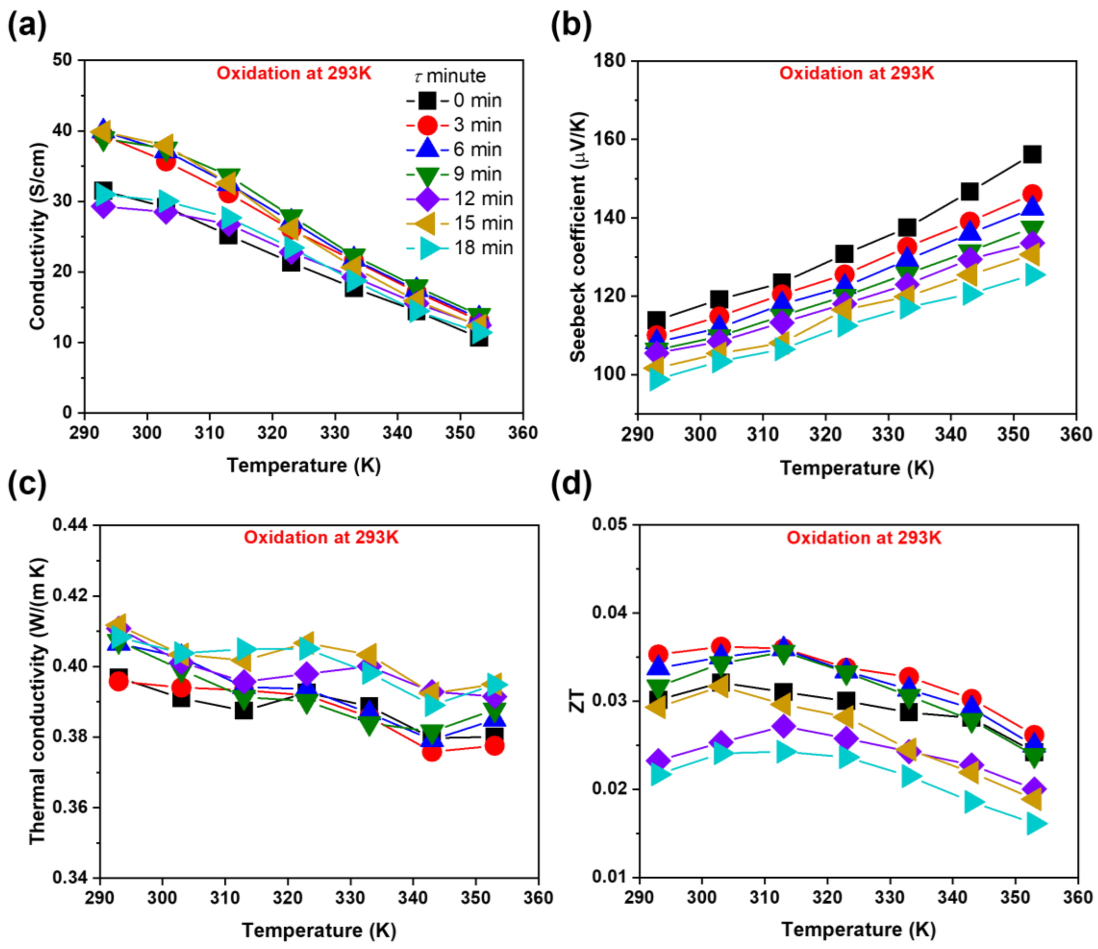

Figure S10. Oxidation (at room temperature) time dependence of thermoelectric properties in $CsSnI_3$ thin films. (a) Electrical conductivity. **(b)** Seebeck coefficient. **(c)** Total thermal conductivity. **(d)** Figure of merit *zT*.

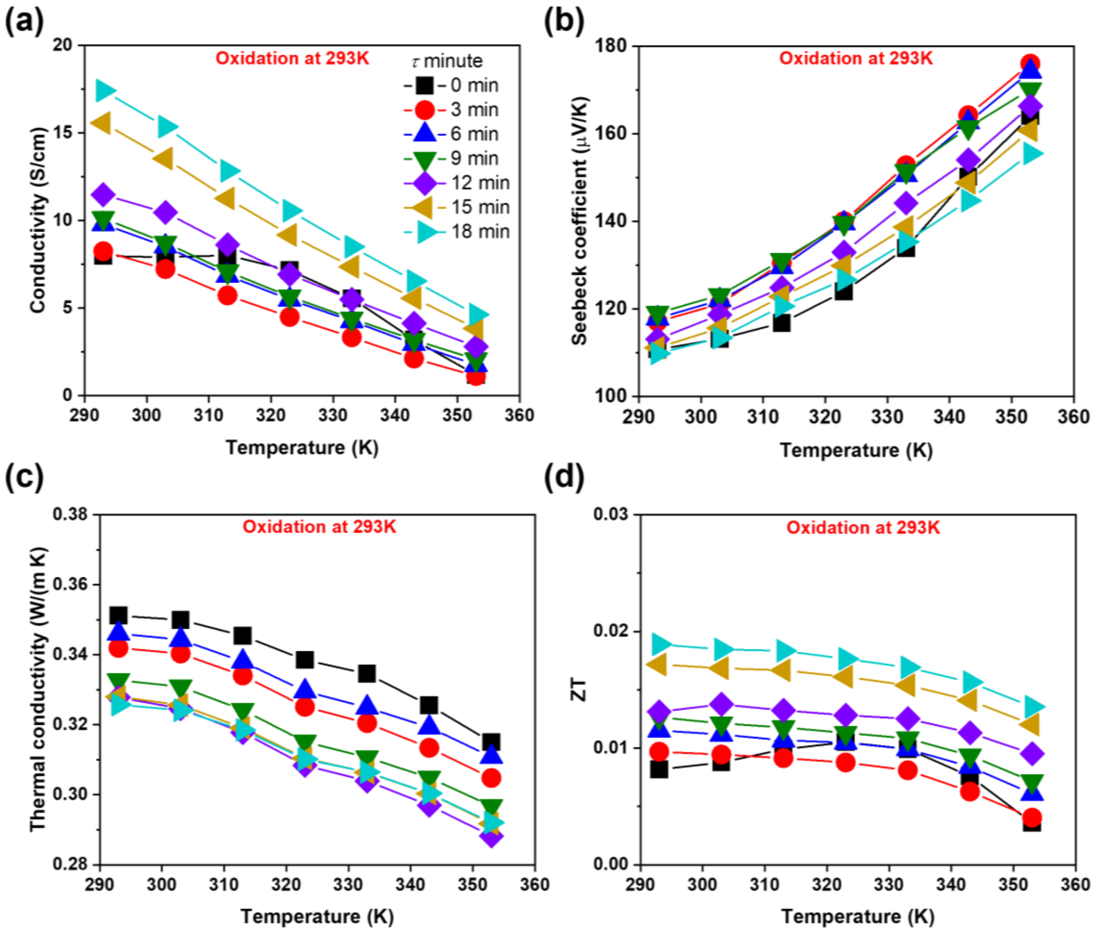

Figure S11. Oxidation (at room temperature) time dependence of thermoelectric properties in $CsSn_{0.95}Ge_{0.05}I_3$ thin films. (a) Electrical conductivity. **(b)** Seebeck coefficient. **(c)** Total thermal conductivity. **(d)** Figure of merit *zT*.

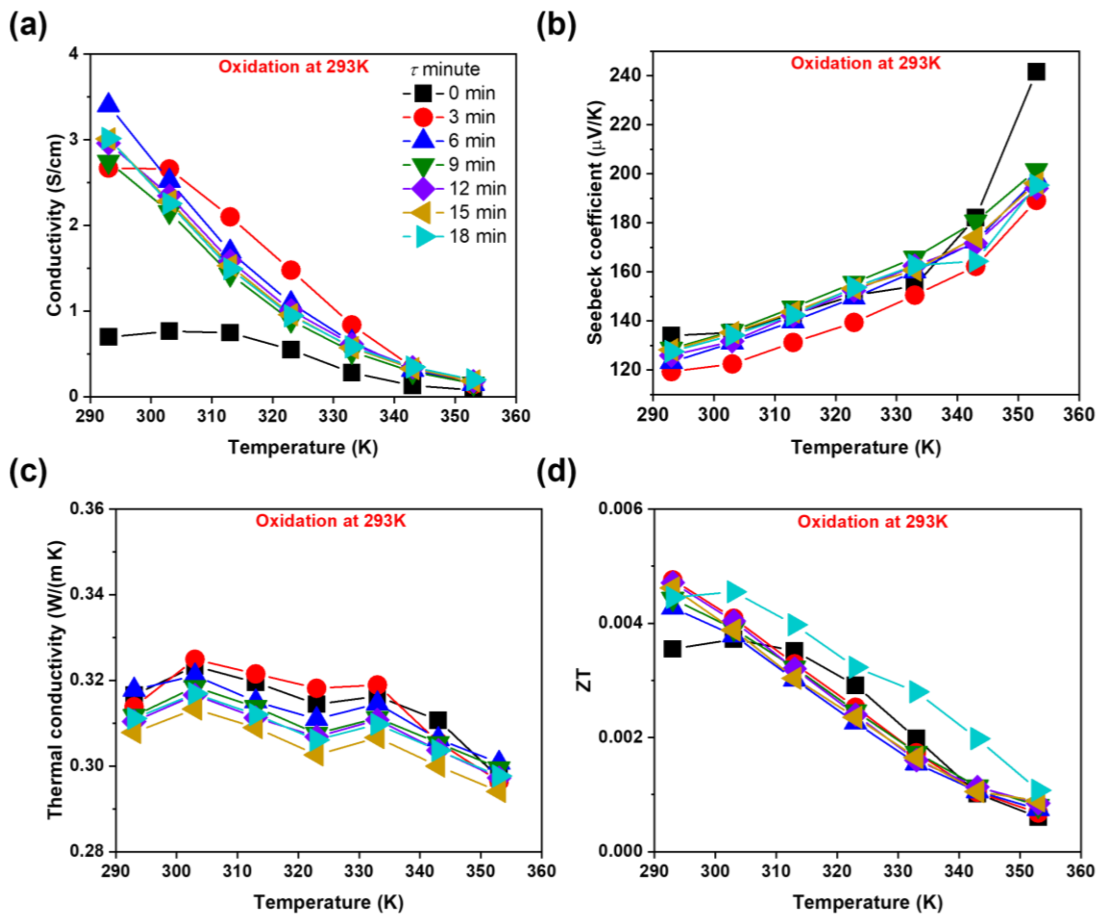

Figure S12. Oxidation (at room temperature) time dependence of thermoelectric properties in $CsSn_{0.9}Ge_{0.1}I_3$ thin films. (a) Electrical conductivity. **(b)** Seebeck coefficient. **(c)** Total thermal conductivity. **(d)** Figure of merit zT.

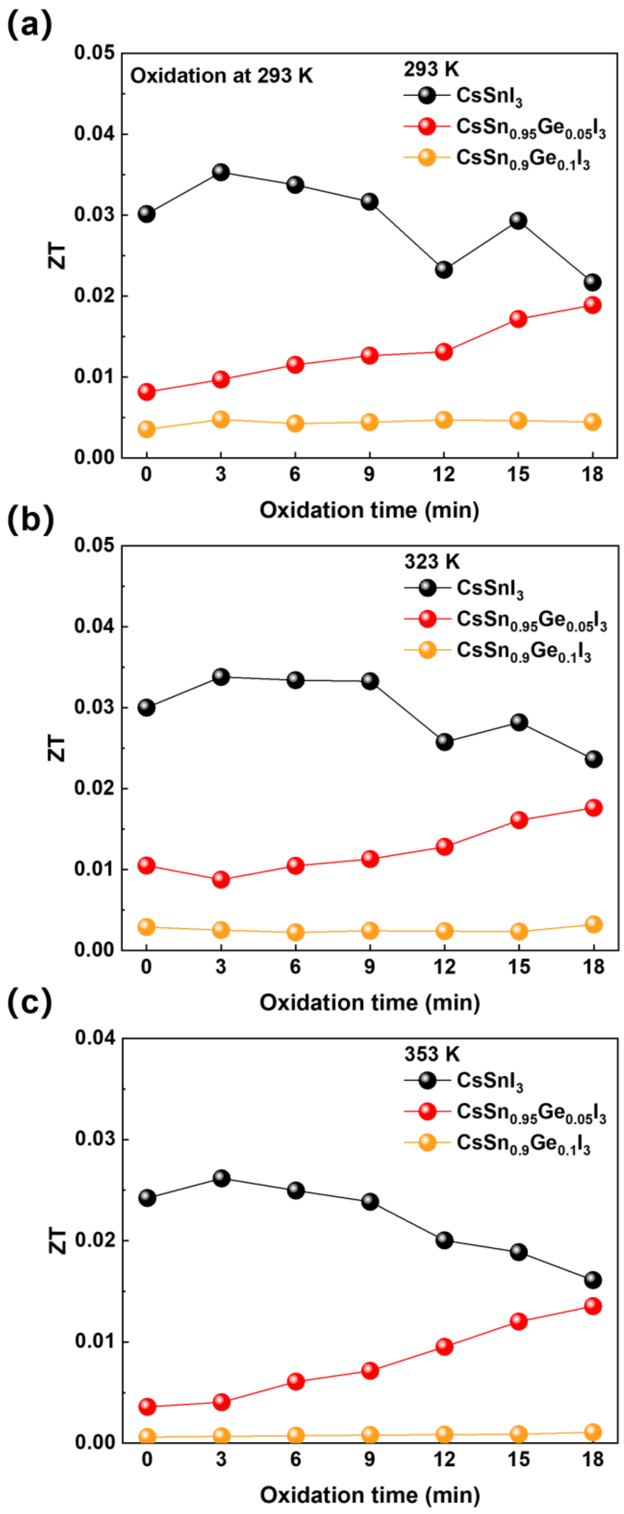

Figure S13. The Figure of merit, zT, stability measurement of mixed $CsSn_{1-x}Ge_xI_3$ films oxidised at 293 K. *zT* is measured at **(a)** 293 K, **(b)** 323 K and **(c)** 353 K.

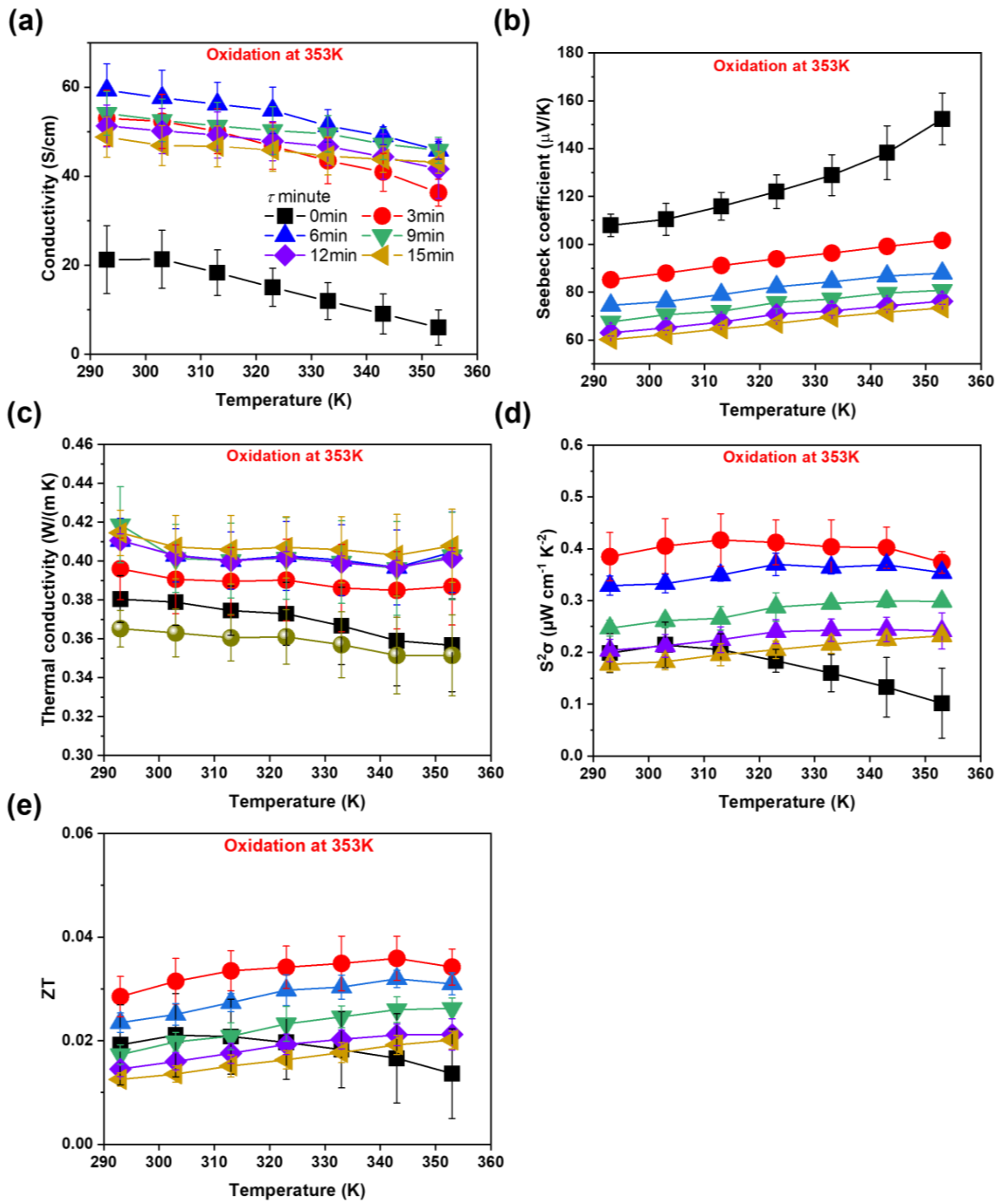

Figure S14. Oxidation (at 80 °C) time dependence of thermoelectric properties in $CsSnI_3$ thin films. **(a)** Electrical conductivity. **(b)** Seebeck coefficient. **(c)** Total thermal conductivity. **(d)** Figure of merit, *zT*.

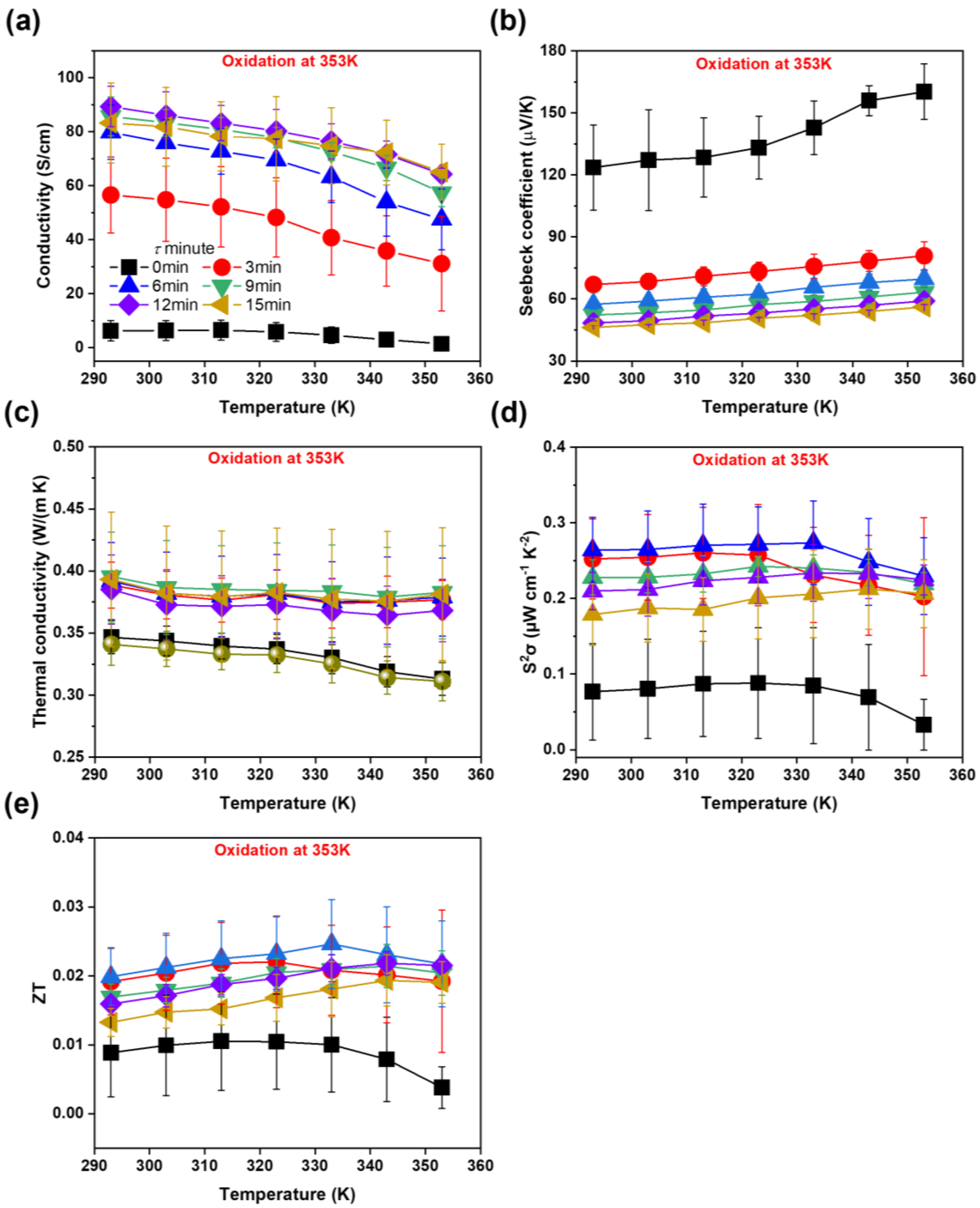

Figure S15. Oxidation (at 80 °C) time dependence of thermoelectric properties in $CsSn_{0.95}Ge_{0.05}I_3$ thin film. (a) Electrical conductivity. **(b)** Seebeck coefficients. **(c)** Total thermal conductivity. **(d)** Figure of merit *zT*.

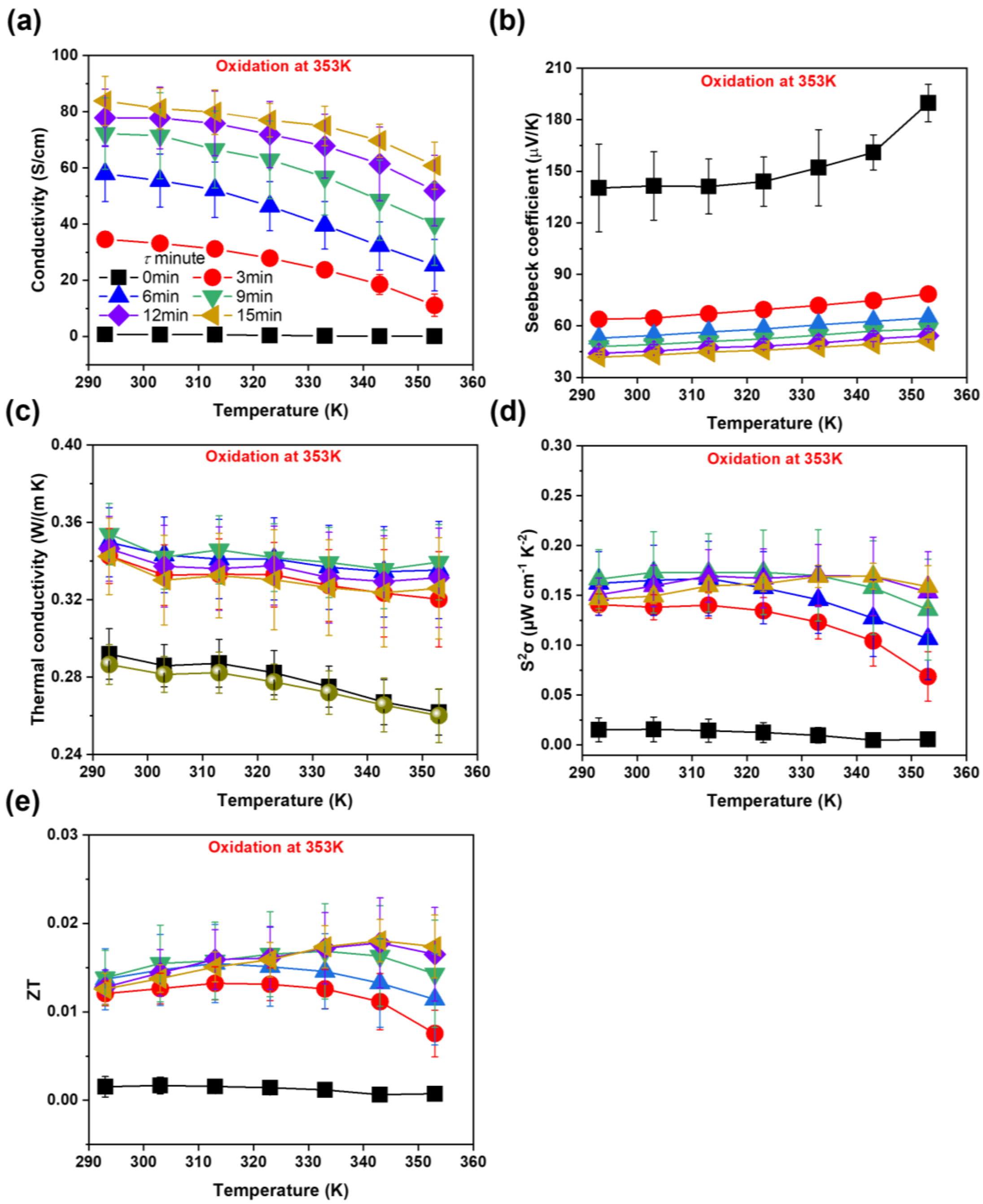

Figure S16. Oxidation (at 80 °C) time dependence of thermoelectric properties in $CsSn_{0.9}Ge_{0.1}I_3$ thin films. (a) Electrical conductivity. **(b)** Seebeck coefficient. **(c)** Total thermal conductivity. **(d)** Figure of merit *zT*.

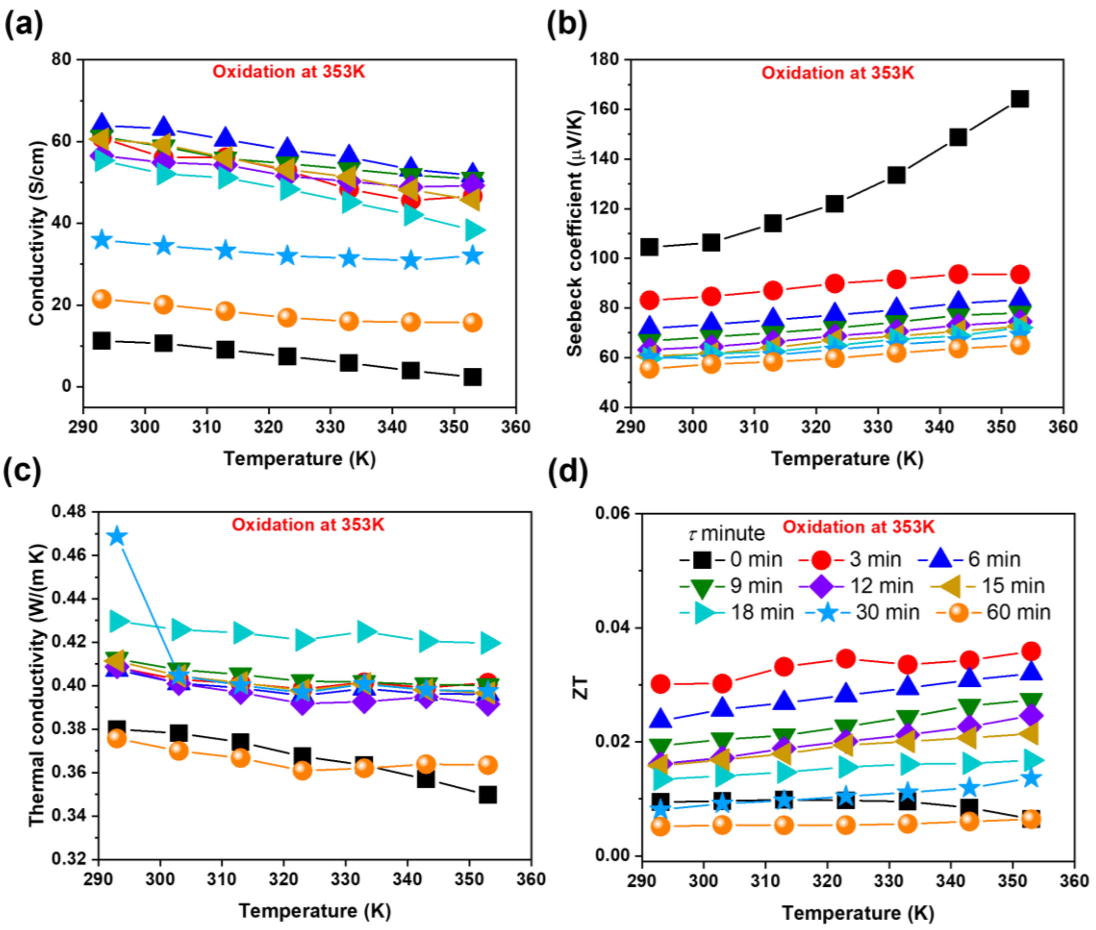

Figure S17. Stability of thermoelectric properties in pristine $CsSnI_3$ thin films (Oxidation at 80 °C). (a) Electrical conductivity. **(b)** Seebeck coefficient. **(c)** Total thermal conductivity. **(d)** Figure of merit *zT*.

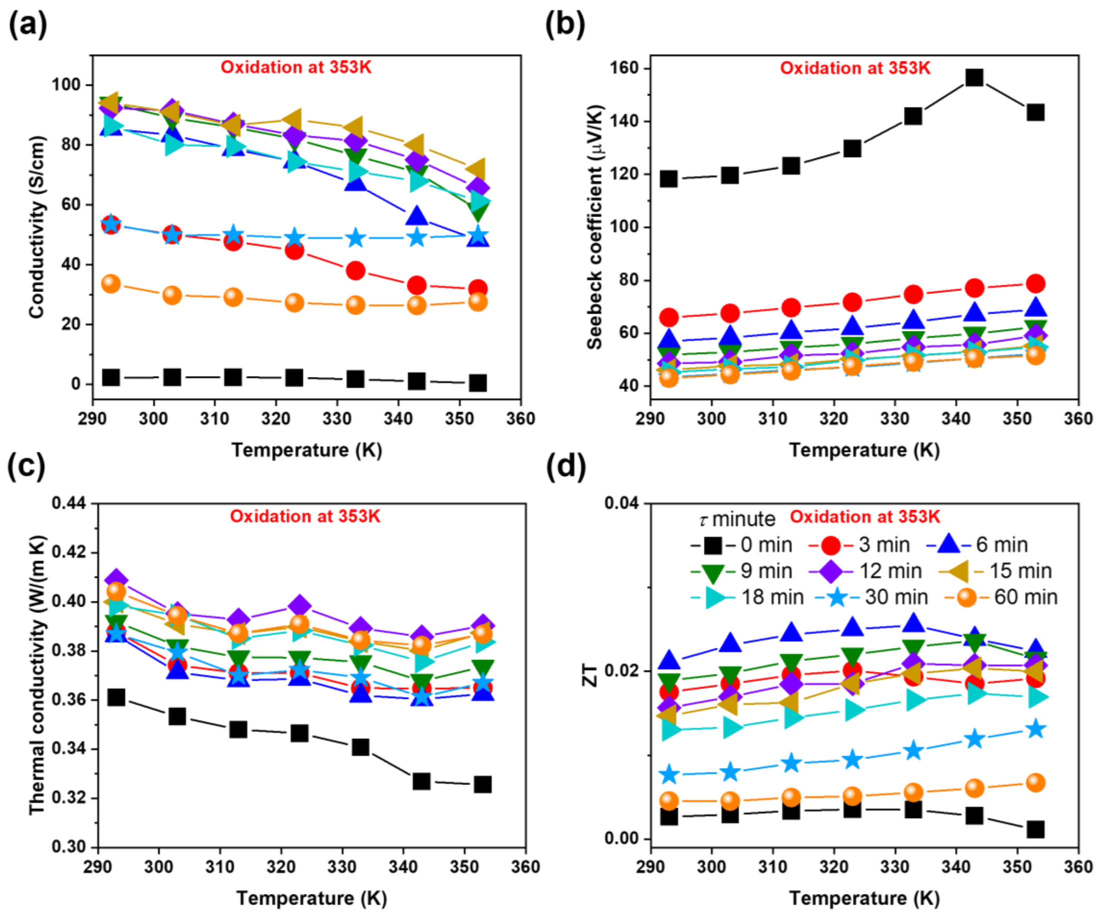

Figure S18. Stability of thermoelectric properties in $CsSn_{0.95}Ge_{0.05}I_3$ thin films (Oxidation at 80 °C). (a) Electrical conductivity. **(b)** Seebeck coefficient. **(c)** Total thermal conductivity. **(d)** Figure of merit, *zT*.

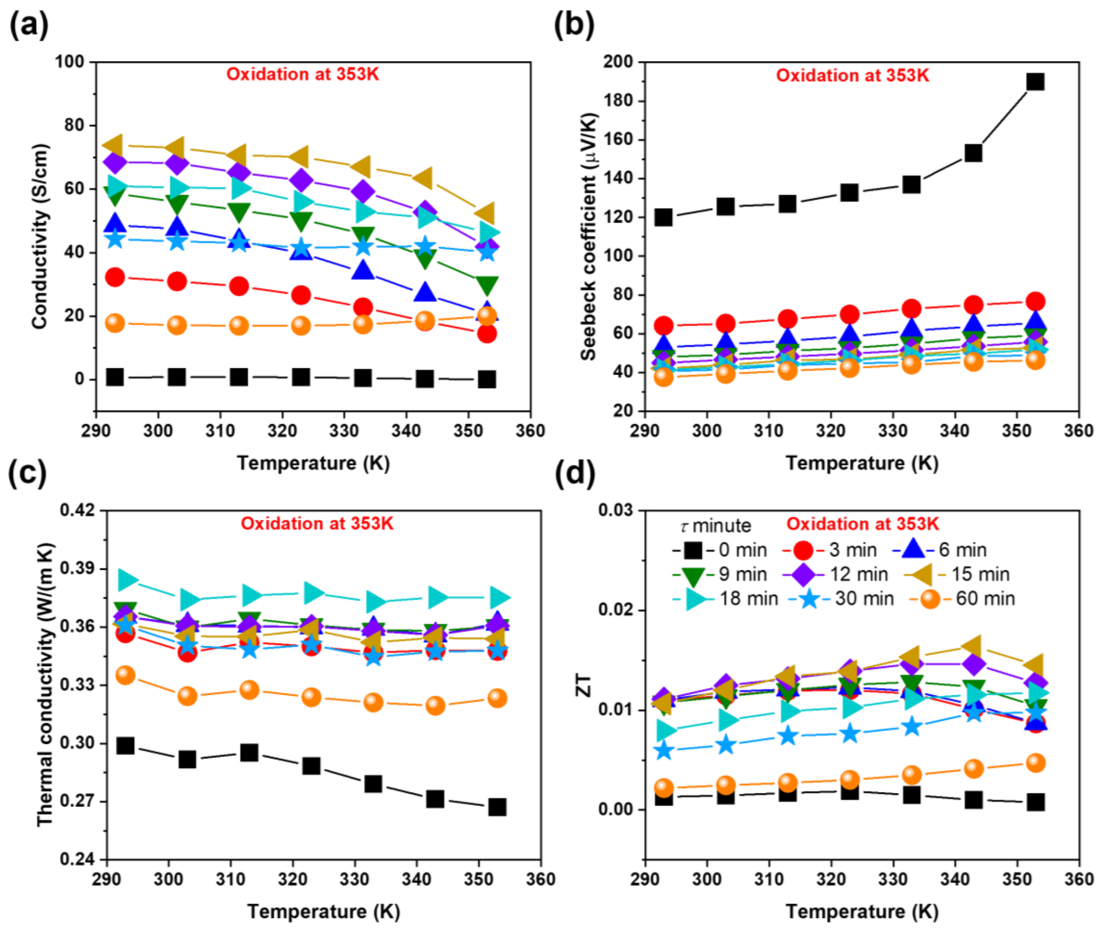

Figure S19. Stability of thermoelectric properties in $CsSn_{0.9}Ge_{0.1}I_3$ thin film (Oxidation at 80 °C). (a) Electrical conductivity. **(b)** Seebeck coefficients. **(c)** Total thermal conductivity. **(d)** Figure of merit, *zT*.

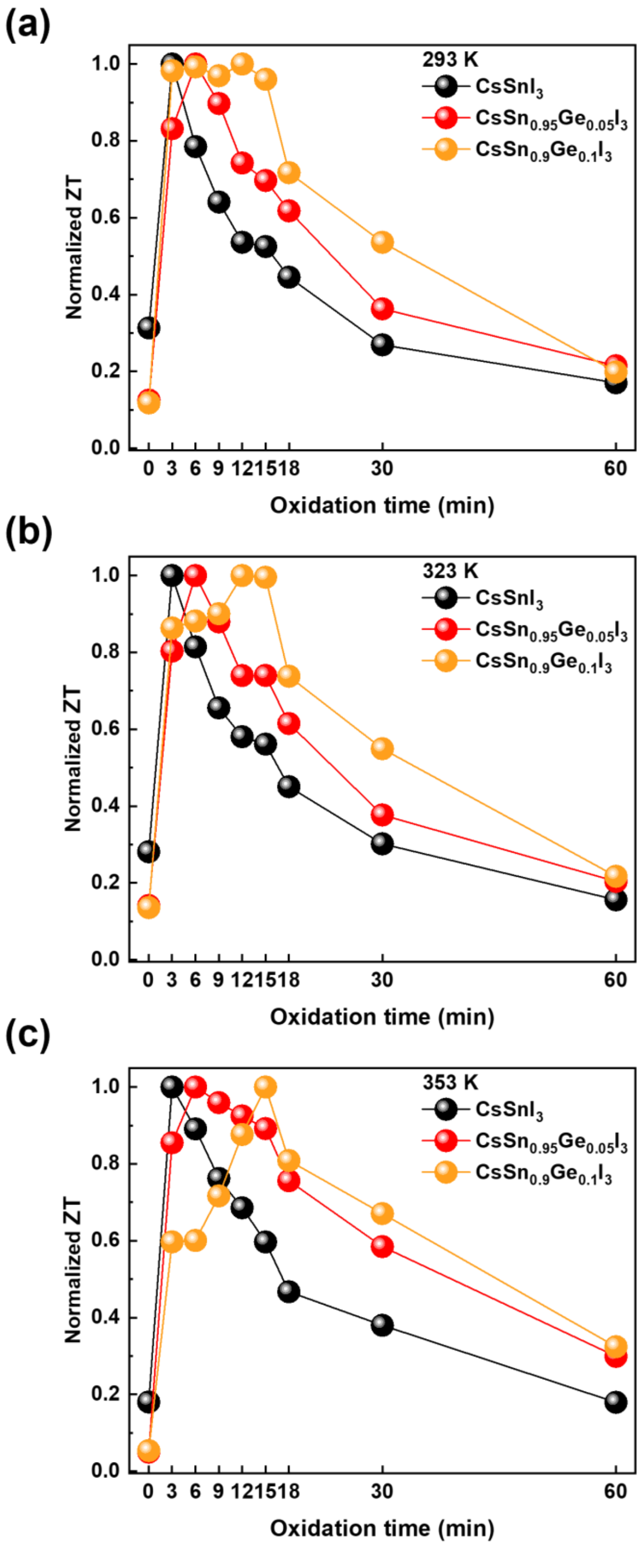

Figure S20. Normalized figure of merit, *zT*, of mixed $CsSn_{1-x}Ge_xI_3$ films as a function of oxidation time recorded at **(a-c)** 293 K, 323 K and 353 K. Oxidation was performed at 353 K.

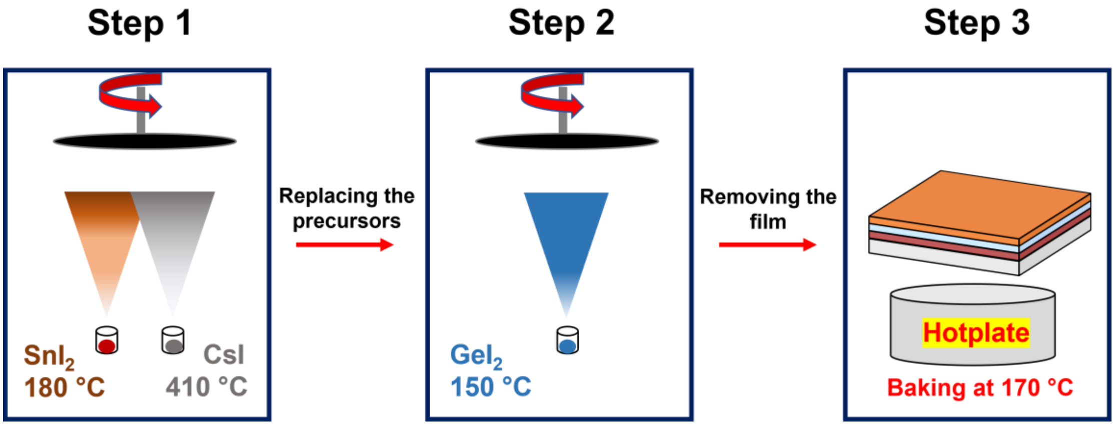

Figure S21. Schematic of the deposition of mixed halide $CsSn_{1-x}Ge_xI_3$ thin films.

The deposition is done in three steps.

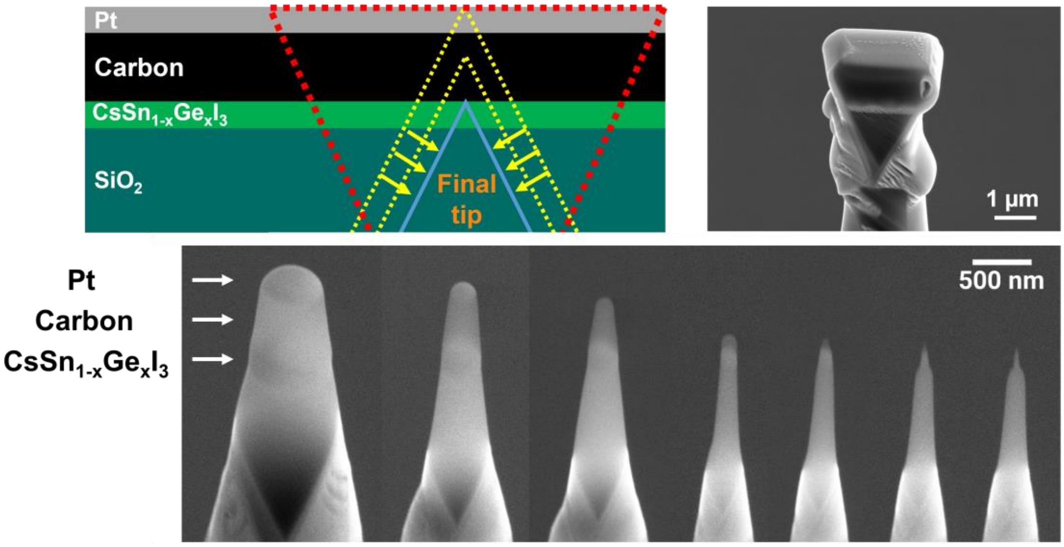

Figure S22. Schematic of atom probe tomography tip preparation with the mixed halide perovskite $CsSn_{1-x}Ge_xI_3$. Red and yellow dots indicate the volumes which are used for lift-off and final milling, respectively.

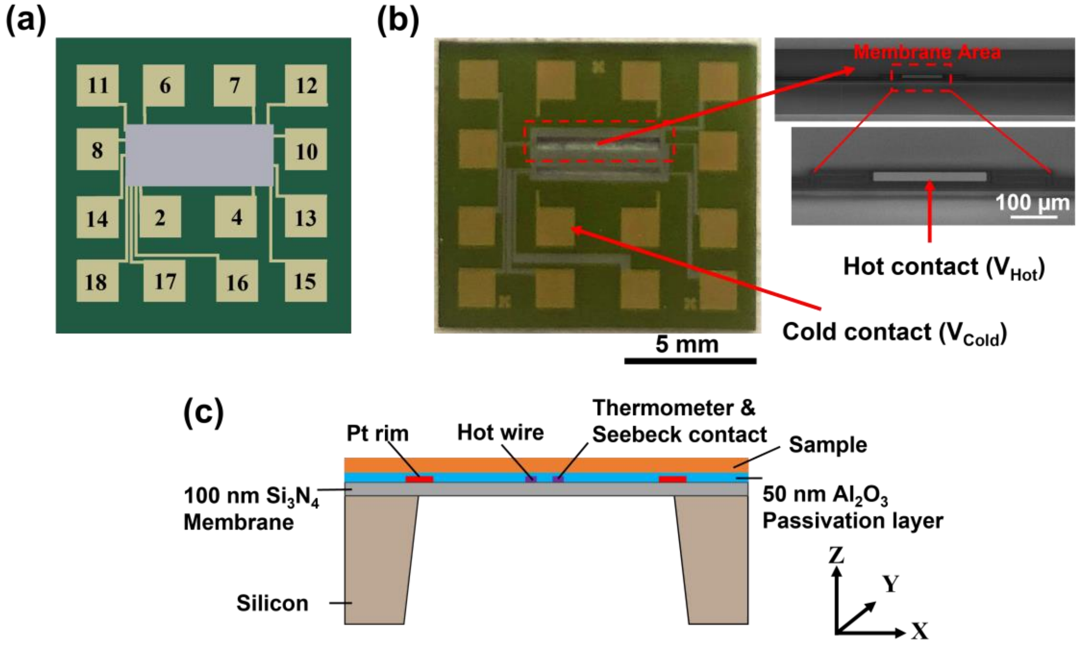

Figure S23. Thermoelectric property measurement of mixed halide perovskite $CsSn_{1-x}Ge_xI_3$ thin film. (a) Schematic of measurement chip with a Van der Pauw setup (perovskite film is deposited on the grey area). (b) Optical image of a measurement chip and SEM image of the heater on the membrane area. (c) Cross-sectional view of the Van der Pauw chip showing the suspended membrane.

Supplemental Notes

Note S1: Thermoelectric property measurement

The resistivity (electrical conductivity) was measured in the van der Pauw configuration using contacts 2, 4, 6, 7 (**Figure S23a**). For the measurement, a current is applied between two contacts (2/6 and 2/4) at one edge and the measured voltage at the other edge is obtained between the remaining two contacts (4/7 and 6/7). The electrical conductivity is calculated by the van der Pauw formula, see equation 1.

$$\exp\left(-\pi d\sigma \times R_{24,67}\right) + \exp\left(-\pi d\sigma \times R_{26,47}\right) = 1 \tag{1}$$

Where d is the thickness, and $R_{ab,cd}$, is the resistance measured from the potential between contacts c and d when a current is applied between contacts a and b. The Seebeck coefficient is measured on the same chip and is calculated using equation 2.

$$S = \frac{-V}{\Delta T} = \frac{-(V_{Hot} - V_{Cold})}{T_{Hot} - T_{Cold}} \tag{2}$$

Where S, V and ΔT are the Seebeck coefficient (V K^{-1}), thermovoltage (V) and temperature gradient (K) between the voltage measurement contacts. V_{Hot}, V_{Cold}, T_{Hot} and T_{Cold} are the electrical potential (V) and absolute temperature (T) at the *hot* and *cold* contacts. For Seebeck coefficient measurement, a current is applied to the heater on the membrane to provide a temperature distribution across the membrane. The Seebeck coefficient is calculated from the gradient of a linear fit of thermal voltage versus ΔT. The total in-plane thermal conductivity of the film-membrane system is measured by using a pseudo-steady state 3Ω technique[1, 2]. The deposited thin film thermal conductivity is extracted using equation 3.

$$\kappa_{measured} * t_{total} = \kappa_S t_S + (\kappa_M t_M + \kappa_P t_P) \tag{3}$$

Where κ and t are thermal conductivity and thickness, respectively. For thermal conductivity measurement, the thermal conductance of the sample ($\kappa_S \times t_S$) is a differential measurement of as-measured total thermal conductance ($\kappa_{measured} \times t_{total}$) and the empty chip (including membrane (M) and passivation layer (P) components: $\kappa_M \times t_M + \kappa_P \times t_P$). The thermal conductance of the membrane plus passivation layer can be calibrated with a zero curve, which comes from the separate measurement of each pristine chip. The cross-sectional view of a pristine chip is depicted in **Figure S23c**. The measurement details are presented in reference[1]. For the oxidation time-dependent thermoelectric property measurement, the as-measured thin film (on a TFA chip) is in-situ oxidised in the measurement chamber for three minutes and then is measured again after evacuating the chamber.

Note S2: Stability of the $CsSn_{1-x}Ge_xI_3$ from DFT calculations

The doping formation energy E_{doping} was defined as:

$$E_{doping} = \frac{1}{x}(E_{\mathrm{CsSn}_{1-x}\mathrm{Ge}_x\mathrm{I}_3} - xE_{Ge} - E_{\mathrm{CsSn}_{1-x}\mathrm{I}_3}) \tag{4}$$

Where $E_{\mathrm{CsSn}_{1-x}\mathrm{Ge}_x\mathrm{I}_3}$, E_{Ge} and $E_{\mathrm{CsSn}_{1-x}\mathrm{I}_3}$ are the system free energy of Ge doping $CsSn_{1-x}Ge_xI_3$, an isolated Ge atom and $CsSn_{1-x}I_3$ with tin vacancy. The DFT calculations were implemented in the Vienna ab initio simulation package (VASP)[3]. The projector augmented wave method (PAW)[4] and generalized gradient approximation (GGA) with the Perdew-Burke-Ernzerhof (PBE) exchange-correlation functional were adopted.[5] The plane-wave cutoff was set to 500 eV and the energy criterion for self-consistent convergence is 1×10^{-6} eV. A 4×4×4 Monkhorst-Pack k-mesh including the Γ point was used to sample the whole Brillouin zone (BZ). The E_{doping} in **Figure S8** are negative, which reflects the thermodynamic stability of Ge-doping into bulk $CsSnI_3$. The negative E_{doping} in **Figure S8** reflects the thermodynamic stability of Ge-doping into $CsSnI_3$ at Sn vacancies.

Note S3: Effect of accelerated oxidation on thermoelectric performance

As elevated temperature can accelerate the self-doping of $CsSnI_3$ thin films and generate holes more rapidly,[6] we performed time-dependent thermoelectric property measurement of $CsSn_{1-x}Ge_xI_3$ films where the in-situ oxidation happens at 80 °C (**Figures S14-S16**). Significant growth of electrical conductivity (**Figure S14a-S16a**)

is observed in all films, increasing from initial values of 21.3 ± 8.0, 6.3 ± 4.0 and 0.7 ± 0.2 S cm^{-1} to maximum values of 59.3 ± 6.0 (6 min), 89.3 ± 8.0 (12 min) and 83.8 ± 9.0 S cm^{-1} (15 min) for $CsSnI_3$, $CsSn_{0.95}Ge_{0.05}I_3$, and $CsSn_{0.9}Ge_{0.1}I_3$, respectively. Correspondingly, the Seebeck coefficient (**Figure S14b-S16b**) of all films experience a dramatical decline, decreasing from 108 ± 5, 124 ± 21 and 140 ± 26 μV K^{-1} to minimum values of 60 ±1, 46 ± 1 and 42 ± 0.6 μV K^{-1} after 15 minutes oxidation. The ratio of S_{15min}/S_{0min} decreases for increasing Ge doping, with values of 0.56 for undoped $CsSnI_3$, 0.37 for $CsSn_{0.95}Ge_{0.05}I_3$ and 0.30 for $CsSn_{0.9}Ge_{0.1}I_3$ films. As the Seebeck coefficient is inversely related to the charge carrier concentration, this trend also supports the idea that Ge substitutions introduce more holes when exposed to air at high temperature despite the increased stability seen in the optical and structural properties caused by Ge-doping.

An increase thermal conductivity (**Figure S14c-S16c)** is observed during oxidation that is attributed to the growing electronic thermal conductivity caused by the hole doping, as reported in our previous work.[6, 7] The maximum thermal conductivity of 0.42 ± 0.02 (pristine $CsSnI_3$), 0.40 ± 0.03 ($CsSn_{0.95}Ge_{0.05}I_3$) and 0.35 ± 0.02 ($CsSn_{0.9}Ge_{0.1}I_3$) W $m^{-1}K^{-1}$ is achieved after 9 minutes oxidation. Finally, the thermoelectric performance (**Figure S14e-S16e**) is improved after air exposure due to the increasing power factor (**Figure S14d-S16d**), resulting in a maximum *zT* of 0.040 ± 0.004 (3 min) at 343 K for undoped $CsSnI_3$, 0.025 ± 0.006 (6 min) at 333 K for $CsSn_{0.95}Ge_{0.05}I_3$ and 0.020 ± 0.002 (15 min) at 343 K for $CsSn_{0.9}Ge_{0.1}I_3$. Furthermore, the one-hour evolution of thermoelectric properties in mixed $CsSn_{1-x}Ge_xI_3$ thin film (oxidation at 80 °C) is shown in **Figure S17-S19**. To investigate the thermoelectric performance stability, we then plot the normalized *zT* (zT/zT_{max}) of mixed $CsSn_{1-x}Ge_xI_3$ films as a function of oxidation time at 293 K, 323 K and 353 K, respectively, see **Figure S20**. Clearly, the

thermoelectric figure merit of pristine $CsSnI_3$ film reaches the peak value at the first 3-minute air exposure and experiences a dramatical reduction during the following air exposure at all temperature, only retaining 20.0% (at 353 K) of the maximum value after 60 minutes of exposure to air. On the contrast, the zT of $CsSn_{0.9}Ge_{0.1}I_3$ films (oxidised at 353 K) undergoes a steady rise in the first 15 minutes and the zT_{60min}/zT_{max} value of 32.4% is obtained during entire air exposure time. Although, the $CsSn_{0.9}Ge_{0.1}I_3$ films present lower zT after oxidation, the better performance stability is observed even oxidising to air at elevated temperature.

Reference

(1) Linseis, V.; Völklein, F.; Reith, H.; Nielsch, K.; Woias, P. Advanced platform for the in-plane ZT measurement of thin films. *Rev. Sci. Instrum.* **2018**, *89* (1), 015110.
(2) Sikora, A.; Ftouni, H.; Richard, J.; Hébert, C.; Eon, D.; Omnès, F.; Bourgeois, O. Highly sensitive thermal conductivity measurements of suspended membranes (SiN and diamond) using a 3ω-Völklein method. *Rev. Sci. Instrum.* **2012**, *83* (5), 054902.
(3) Kresse, G.; Furthmüller, J. Efficient iterative schemes for ab initio total-energy calculations using a plane-wave basis set. *Phys. Rev. B* **1996**, *54* (16), 11169-11186. Kresse, G.; Furthmü ller, J. Efficiency of ab-initio total energy calculations for metals and semiconductors using a plane-wave basis set. *Comput. Mater. Sci.* **1996**, *6* (1), 15-50.
(4) Blöchl, P. E. Projector augmented-wave method. *Phys. Rev. B* **1994**, *50* (24), 17953-17979. Kresse, G.; Joubert, D. From ultrasoft pseudopotentials to the projector augmented-wave method. *Phys. Rev. B* **1999**, *59* (3), 1758-1775.
(5) Perdew, J. P.; Burke, K.; Ernzerhof, M. Generalized Gradient Approximation Made Simple. *Phys. Rev. Lett.* **1996**, *77* (18), 3865-3868.
(6) Tang, W.; Liu, T.; Fenwick, O. High thermoelectric performance based on $CsSnI_3$ thin films with improved stability. *J. Mater. Chem. A* **2022**, *10* (13), 7020-7028.
(7) Liu, T.; Zhao, X.; Li, J.; Liu, Z.; Liscio, F.; Milita, S.; Schroeder, B. C.; Fenwick, O. Enhanced control of self-doping in halide perovskites for improved thermoelectric performance. *Nat. Commun.* **2019**, *10* (1), 5750.
